# Optimization of Pore-Space-Partitioned Metal–Organic Frameworks Using the Bioisosteric Concept

**DOI:** 10.1021/jacs.2c09349

**Published:** 2022-10-28

**Authors:** Huajun Yang, Yichong Chen, Candy Dang, Anh N. Hong, Pingyun Feng, Xianhui Bu

**Affiliations:** † Department of Chemistry and Biochemistry, California State University, Long Beach, California 90840, United States; ‡ Department of Chemistry, University of California, Riverside, California 92521, United States

## Abstract

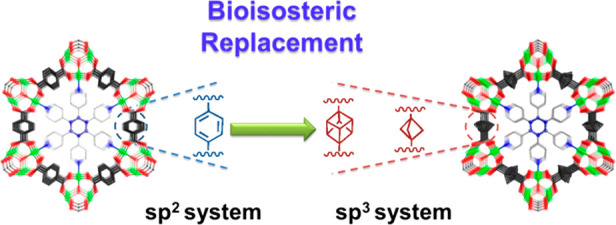

Pore space partitioning (PSP) is methodically suited for dramatically increasing the density of guest binding sites, leading to the partitioned acs (pacs) platform capable of record-high uptake for CO_2_ and small hydrocarbons such as C_2_H_*x*_. For gas separation, achieving high selectivity amid PSP-enabled high uptake offers an enticing prospect. Here we aim for high selectivity by introducing the bioisosteric (BIS) concept, a widely used drug design strategy, into the realm of pore-space-partitioned MOFs. New pacs materials have high C_2_H_2_/CO_2_ selectivity of up to 29, high C_2_H_2_ uptake of up to 144 cm^3^/g (298 K, 1 atm), and high separation potential of up to 5.3 mmol/g, leading to excellent experimental breakthrough performance. These metrics, coupled with exceptional tunability, high stability, and low regeneration energy, demonstrate the broad potential of the BIS-PSP strategy.

Because of the prevalence of aromatic rings in small-molecule drugs, bioisosteric replacement (BIS), commonly practiced by replacing benzene rings with other scaffolds, has become an important method in drug design.^1^ Given the similar prevalence of aromatic rings in framework materials (e.g., metal–organic frameworks (MOFs), covalent organic frameworks, and hydrogen-bonded organic frameworks), leveraging the BIS strategy for framework materials design has the potential to further vitalize their development.^2−6^ There have been sporadic examples in which benzene rings were replaced by aliphatic moieties on platforms including MOF-5 and UiO-66.^7−9^ However, the purposeful design of framework materials leveraging the BIS strategy and its large toolbox and database has yet to start.^10−13^

The application of the BIS strategy in framework materials faces an extra hurdle because in addition to the design of molecular bioisosteres, the additional step of framework formation poses a challenge for bioisostere incorporation. With this in mind, we aim to identify platforms with a built-in structure-directing effect that can guide bioisosteres into intended structures. Here, by integrating pore space partitioning (PSP) with the BIS strategy (Scheme 1), we show that partitioned acs (pacs) is an ideal platform for leveraging the BIS strategy due to synergistic effects among its modules, ultrahigh chemical and geometrical tunability, and high stability.^14,15^

**Scheme 1:**
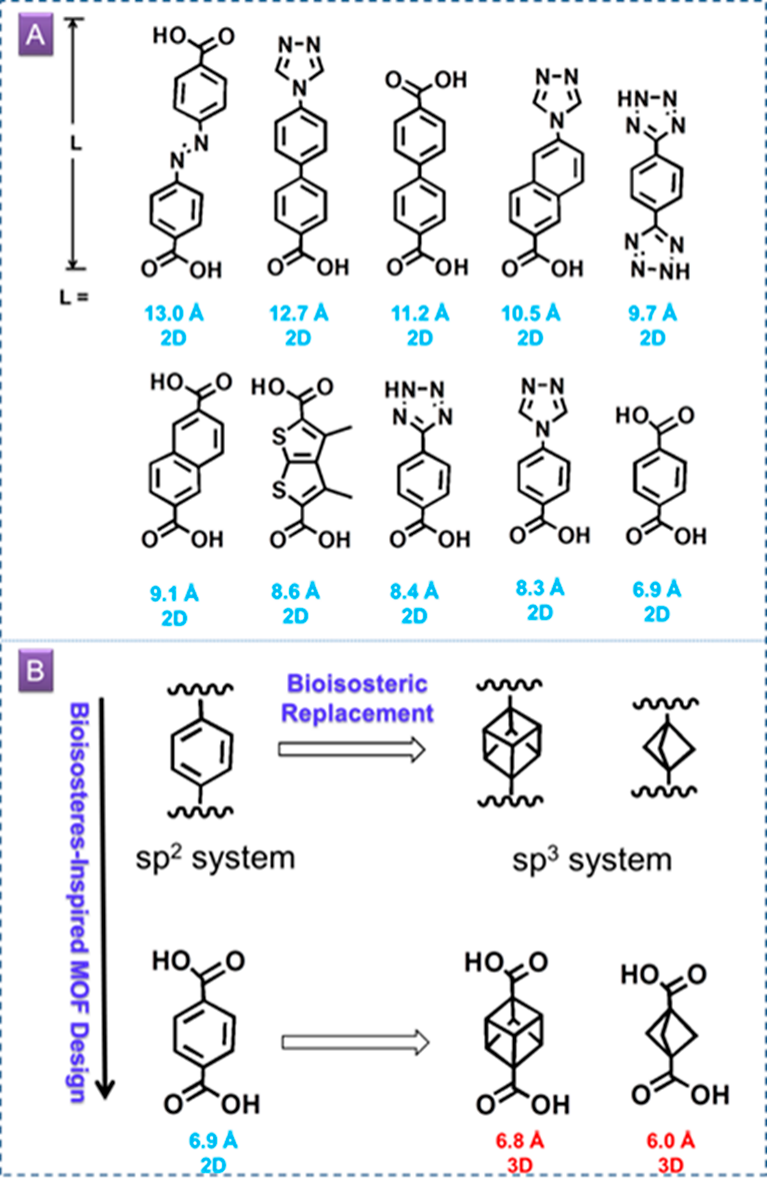
(A) Size Range of sp^2^-L1 Ligands in Pacs (Ligands Are Shown with Substituents Omitted); (B) BIS Strategy in Drug Design and Its Parallel in Framework Materials Design

The pacs platform is built from a pore-partitioning agent (denoted L2) and the acs-type MIL-88/MOF-235 framework made from ligand L1 and metal trimers. Of relevance to the BIS strategy is the observed strong interdependent structure-directing effects between L1–trimer and L2–trimer formation.^16−18^ As a result, the pacs platform is exceptionally accommodative of variations in L1 or L2 (and in the trimers as well) and therefore provides unparalleled opportunities for implementing the BIS strategy. It should be noted that the BIS strategy is best applied to edit chemical systems that already show promising properties for targeted applications. Such is the case for pacs because the PSP strategy, intrinsic to pacs platform, has led to near- or at-record-high gas (e.g., C_2_H_*x*_) uptake capacities.^19−21^ With such well-established uptake and high stability, we are intrigued by the prospect that the integration of BIS and PSP may offer a solution to yet another critical parameter in gas separation: selectivity.

To date, L1 ligands in reported pacs are sp^2^-based, with the smallest L1 being terephthalate (bdc). In medicinal chemistry, it has been discovered that bioisosteric replacement using bioisosteres with increased 3-D character can lead to decreased nonspecific binding. This effect can be similarly explored in MOFs to help improve gas selectivity. The ligand 3-D character can be measured with the *F*_sp^3^_ value (the ratio of the number of sp^3^ carbons to the total number of carbons). In the past decade, the pursuit of 3-D cyclic scaffolds in drug design has led to the wide use of bicyclo[1.1.1]pentane (bcp) as a bioisostere for para-substituted benzene rings.^22,23^ In addition to its 3-D character, bcp is extreme because it is the smallest bridged bicyclic ring (only ∼1.87 Å between bridgehead carbons, compared with 2.79 Å between the para positions in benzene).

In this work, bicyclo[1.1.1]pentane-1,3-dicarboxylic acid (H_2_bcp) and cubane-1,4-dicarboxylic acid (H_2_cdc) were used to build pacs using the BIS-PSP strategy. Among sp^3^ bioisosteres, bcp (C_5_H_8_), cubane (C_8_H_8_), and bicyclo[2.2.2]octane (C_8_H_14_) are among the best for their match with the para substitution pattern of the benzene ring. They are able to serve the same scaffolding role as the benzene ring and yet engage in different electronic and steric interactions with targets. Two series of pacs materials are reported here: CPM-111 from bcp and CPM-125 from cdc. Ultramicropores (∼5.9 Å) were achieved with bcp, together with multifold enhancement of the C_2_H_2_/CO_2_ and C_3_H_6_/C_3_H_8_ selectivities. CPM-111a-Ni (i.e., Ni_3_-bcp-tpt) is an excellent adsorbent in terms of key separation metrics, including high selectivity, high gravimetric and volumetric uptake, easy regeneration, and high stability.

Even with just bcp and cdc as L1 ligands, many new pacs materials are accessible using diverse trimers and L2 ligands. Here we synthesized nine representative materials from five types of trimers (Ni_3_, Co_3_, Mg_3_, Co_*x*_V_3–*x*_, and Ni_*x*_V_3–*x*_) and two types of L2 ligands (tpt = 2,4,6-tris(4-pyridyl)-1,3,5-triazine) and tppy = 2,4,6-tris(4-pyridyl)pyridine). Pure Ni_3_-bdc-tpt (CPM-33a) was synthesized for comparison (Tables S1–S5).

The bcp family expands the known lower limit of the L1/L2 length ratio and the related unit-cell *c*/*a* ratio. These ratios are important parameters of the pacs platform and control the pore shape and size. All new pacs materials have nearly the same *a* length (∼16.9 Å) as bdc-pacs because the *a* axis is primarily determined by L2. However, the *c* length is significantly reduced in bcp-pacs (from 15.2 to 12.1 Å), leading to a *c*/*a* ratio of 0.71, the lowest reported to date.^24^

Bioisosteric replacement in pacs (bdc → cdc → bcp) leads to stepwise control over the pore size and pore surface area. The cdc ligand is slightly shorter than bdc (2.72 Å between the bridgehead carbons in cdc vs 2.79 Å between the para positions in benzene) but has a 3-D shape with two extra CH groups. The pore dimensions measured between metal trimer nodes barely change in going from bdc to cdc, but the accessible pore space is smaller due to the sp^3^ L1 ligand. In particular, while the height of the hexagonal pores shows only a slight decrease (4.5 to 4.3 Å) in going from bdc to cdc, the inner radius of the trigonal-bipyramidal pores decreases from 4.5 to 3.2 Å. In going from bdc to bcp, both types of pores are compressed significantly along the *c* axis. The height of the hexagonal pores is reduced to 2.9 Å, a 35% decrease from bdc-pacs, while the radius of the trigonal-bipyramidal pores decreases to 3.0 Å (Figure 1). Correspondingly, the guest-accessible volume ratio calculated from PLATON decreases from 58.1% in Ni_3_-bdc-tpt to 53.5% in Ni_3_-cdc-tpt and 33.0% in Ni_3_-bcp-tpt.

**Figure 1:**
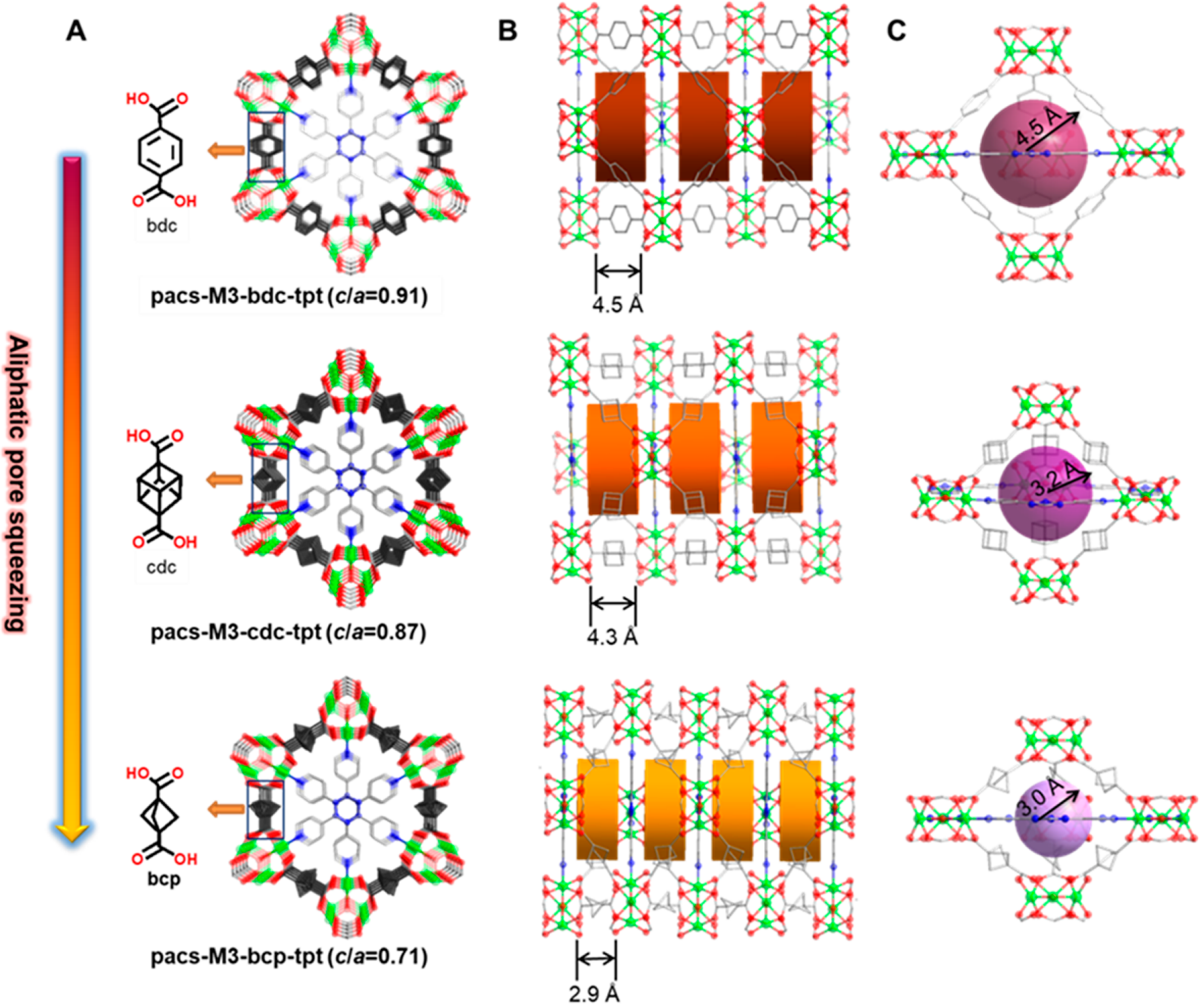
Comparison between pacs-M_3_-L1-tpt materials (L1 = bdc, cdc, bcp). (A) Views along the *c* axis. The *c*/*a* ratios were calculated from the Ni_3_ compositions. (B) Side views of hexagonal cylinder pores. (C) Side views of trigonal-bipyramidal pores. Color code: green, Ni; red, O; blue, N; gray, C. The distances in (B) and (C) exclude the van der Waals radii of surface atoms.

The pore shrinkage in going from bdc to cdc and bcp was confirmed by isotherms (Figure 2A). DFT-calculated pore size distributions showed that pore sizes of Ni_3_-cdc-tpt and Ni_3_-bcp-tpt are centered around 5.9 Å (ultramicropores). This is in contrast to 6.8 Å for Ni_3_-bdc-tpt. The short and bulky bcp also leads to a significant reduction in Brunauer–Emmett–Teller (BET) surface area (Table S6).

**Figure 2:**
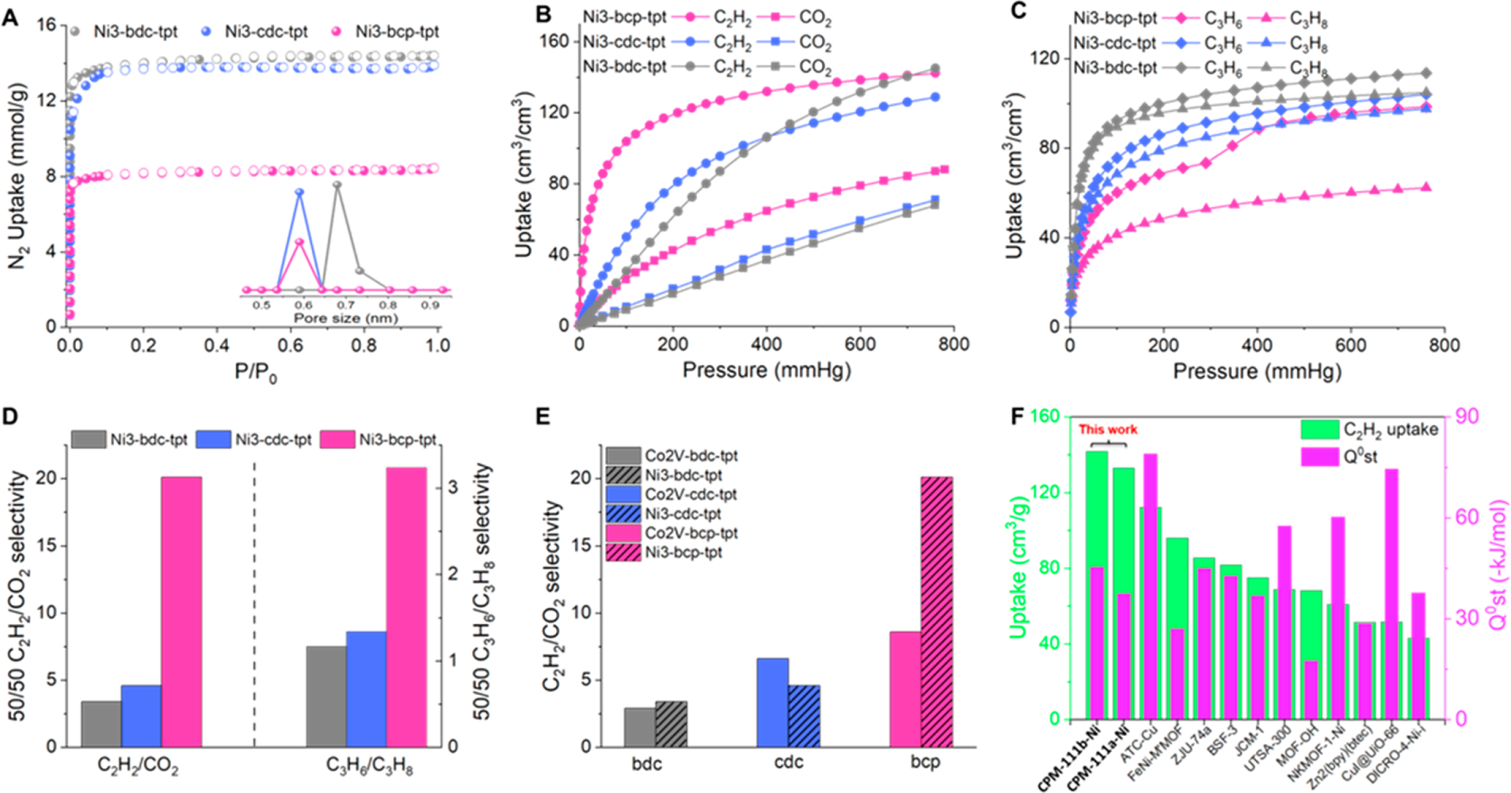
Various comparisons of (A) N_2_ adsorption isotherms and DFT pore size distributions, (B) C_2_H_2_ and CO_2_ adsorption isotherms at 298 K, (C) C_3_H_6_ and C_3_H_8_ adsorption isotherms at 298 K, (D) 50/50 C_2_H_2_/CO_2_ and C_3_H_6_/C_3_H_8_ selectivities for different L1 ligands, (E) C_2_H_2_/CO_2_ selectivities between Ni_3_ and Co_2_V trimers, and (F) C_2_H_2_ uptakes (298 K and 1 atm) and *Q*_st_^0^ values for top-performing MOFs with C_2_H_2_/CO_2_ selectivity > 12.

The adsorption properties of two gas pairs (C_2_H_2_/CO_2_ and C_3_H_6_/C_3_H_8_) were studied (Figures S5–S12). These pacs materials showed efficient C_2_H_2_/CO_2_ separation performance (Figure 2B). The gravimetric C_2_H_2_ uptakes at 298 K and 1 atm were 133.0, 130.7, and 162.1 cm^3^/g for Ni_3_-bcp-tpt, Ni_3_-cdc-tpt, and Ni_3_-bdc-tpt, respectively, corresponding to volumetric uptakes of 142.3, 128.7, and 144.9 cm^3^/cm^3^. Despite the smaller size of bcp, the decrease in C_2_H_2_ uptake is minor in going from Ni_3_-bdc-tpt to Ni_3_-bcp-tpt and is negligible for the volumetric uptake. This indicates more efficient pore use and a higher packing density of C_2_H_2_ in Ni_3_-bcp-tpt. The C_2_H_2_ packing density of Ni_3_-bcp-tpt is 0.5 g/cm^3^. This value is among the highest in MOFs and is 73% higher than that of Ni_3_-bdc-tpt (0.29 g/cm^3^).^25−27^ It should be noted that the actual packing density could be higher because the pore volume is overestimated due to omission of guest cations in the calculation (Figures S13 and S14).

The improvement in the C_3_H_6_/C_3_H_8_ separation in going from bdc and cdc to bcp is profound (Figure 2C). Ni_3_-bdc-tpt and Ni_3_-cdc-tpt could barely separate them due to the similar C_3_H_6_ and C_3_H_8_ isotherms. In comparison, there is a big gap between the C_3_H_6_ and C_3_H_8_ adsorption isotherms for Ni_3_-bcp-tpt. Also interesting is the observation of flexible-robust behavior in the C_3_H_6_ adsorption isotherm with an uptake jump at around 300 mmHg.^28,29^ This gating behavior could be related to molecular dynamics of bcp such as rotation of the bicyclic ring upon target binding.^11,30^

Ideal adsorbed solution theory (IAST) selectivity calculations confirmed the dramatic enhancement upon bioisosteric replacement with bcp (Figure 2D). Ni_3_-bcp-tpt shows a high C_2_H_2_/CO_2_ selectivity of 20.1 at 298 K and 1 bar, about 6 times that of its bdc counterpart. There is also a 177% increase in C_3_H_6_/C_3_H_8_ selectivity from Ni_3_-bdc-tpt to Ni_3_-bcp-tpt. Notably, the C_2_H_2_/CO_2_ selectivity for Ni_3_-bcp-tpt is higher than those for many top-performing MOFs, such as JCM-1 (13.7), DICRO-4-Ni-i (13.9), and BSF-3 (16.3), and is especially remarkable among MOFs with high C_2_H_2_ uptake.^31−33^ The higher performance of the materials based on sp^3^ ligands is likely due to the smaller pores and the extra hydrogen atoms on the ligand surface, which can provide more interaction sites, especially for gases that can act as hydrogen acceptors (e.g., C_2_H_2_ with C^δ−^).

Significantly, the ultramicropore environment of bcp-based frameworks amplifies the impact of other structural variations on the separation performance. In going from the neutral Co_2_V-based framework to the anionic Ni_3_-based framework, the C_2_H_2_/CO_2_ selectivities are comparable for bdc- and cdc-based pacs. However, there is a dramatic improvement in selectivity (134% increase) in going from Co_2_V-bcp-tpt to Ni_3_-bcp-tpt (from 8.6 to 20.1). Changing Ni to Mg also leads to a dramatic 44% increase in the selectivity from 20.1 to 29.0 (Figure 2E). Furthermore, while the replacement of tpt by tppy shows little influence on the separation performance in bdc-pacs, it has a significant impact on the C_2_H_2_/CO_2_ selectivity in bcp-pacs (Table S7).

To highlight the success of the BIS-PSP strategy, we compare the high selectivities and high uptakes of the new bcp-based pacs materials with those of top-performing MOFs. Among MOFs with high C_2_H_2_/CO_2_ selectivity (>12), Ni_3_-bcp-tpt and Ni_3_-bcp-tppy likely have the highest C_2_H_2_ uptakes at both 0.1 bar and 1 atm (Figure 2F).^34−36^ Among MOFs with higher or comparable C_2_H_2_ uptakes for C_2_H_2_/CO_2_ separation, Ni_3_-bcp-tpt has much higher selectivity (Table S8). The separation potential, which is a metric incorporating the influence of both selectivity and uptake, is used to evaluate separation performance.^37,38^ The bcp pacs materials show very high separation potentials ranging from 4.4 to 5.3 mmol/g. The pacs-Mg_3_-bcp-tpt material has the highest separation potential in this series, 5.3 mmol/g, which is higher than those of previous benchmark pacs MOFs (FJU-90 and SNNU-27).^39−42^ The breakthrough experiments showed that Ni_3_-bcp-tpt had a long breakthrough time and excellent separation performance (Figure S15).

The bcp pacs materials also feature a low adsorption enthalpy, which is highly desirable due to reduced energy consumption for regeneration. The isosteric heat of adsorption at near-zero coverage (*Q*_st_^0^), calculated from adsorption isotherms at 273 and 298 K, is 37.5 kJ/mol for Ni_3_-bcp-tpt. This value is quite small among MOFs with high C_2_H_2_/CO_2_ selectivity, such as ATC-Cu (79.1 kJ/mol) and NKMOF-1 (60.3 kJ/mol).^43−46^ The easy regeneration of Ni_3_-bcp-tpt was confirmed by multiple cycles of gas adsorption experiments. The framework was found to show no capacity loss in five cycles and needed only mild reactivation conditions (60 °C for 30 min) (Figure S16).

In addition to excellent sorption performance, Ni_3_-bcp-tpt has high chemical stability compared with aromatic Ni_3_-bdc-tpt as well as other top-performing MOFs for C_2_H_2_/CO_2_ separation. Powder X-ray diffraction confirmed that Ni_3_-bcp-tpt maintained its crystallinity after being soaked in water for 24 h. In comparison, diffraction peak broadening was observed in Ni_3_-bdc-tpt (Figure S17). The stability difference was further shown by gas adsorption. N_2_ adsorption at 77 K showed a large decrease in surface area for Ni_3_-bdc-tpt after water treatment as well as a defect-related hysteresis loop (Figure S18). In comparison, there was only minor surface area decrease for Ni_3_-bcp-tpt. C_2_H_2_ adsorption confirmed the stability difference observed in N_2_ adsorption. Water treatment caused a 47.3% loss of C_2_H_2_ uptake for Ni_3_-bdc-tpt, while the loss was only 7.7% for Ni_3_-bcp-tpt (Figure 3).

**Figure 3:**
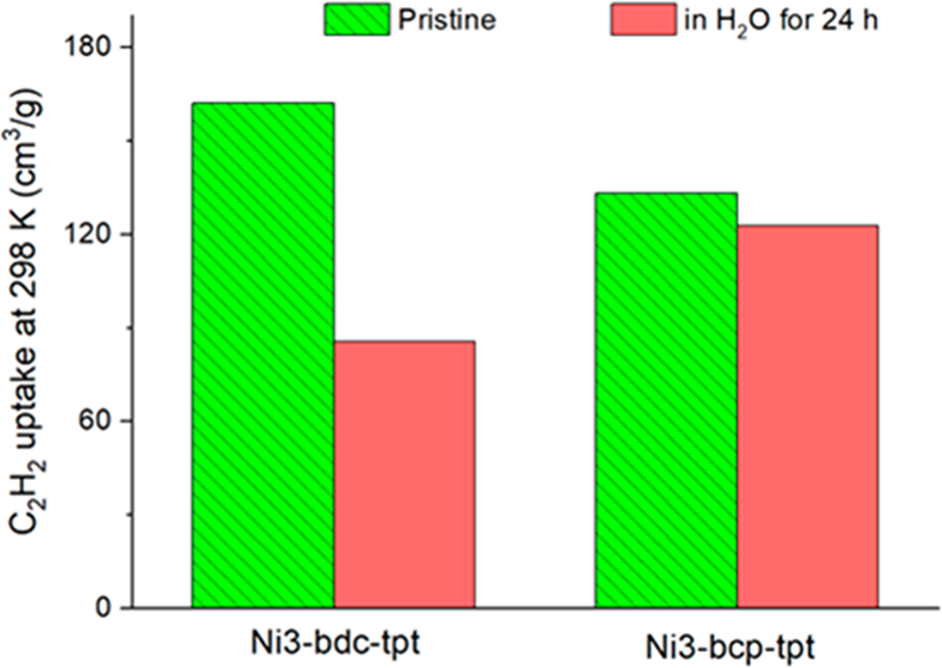
Comparison of hydrothermal stability between Ni3-bdc-tpt and Ni3-bcp-tpt based on C_2_H_2_ uptake at 298 K.

In conclusion, by integrating the bioisosteric replacement strategy with pore space partitioning, we successfully synthesized a family of ultramicroporous materials with much-enhanced gas separation properties and chemical stability. While this work has already demonstrated high gas separation potentials, the generality of the BIS-PSP method promises even further optimization of other MOF platforms as well as other types of framework materials.

## Supporting Information Available

Experimental procedures and compound characterization data (PDF)
