# Supplementary material for: Optimization of Pore-Space-Partitioned Metal–Organic Frameworks Using the Bioisosteric Concept
Source: J Am Chem Soc. 2022 Oct 28;144(44):20221–6. doi: 10.1021/jacs.2c09349 (PMC9650692; doi:10.1021/jacs.2c09349)
Supplement: Supplementary file 1 — ja2c09349_si_001.pdf [file ja2c09349_si_001.pdf]

Optimization of Pore-Space-Partitioned Metal-Organic Frameworks Using Bioisosteric Concept

Huajun Yang,[a,b] Yichong Chen,[b] Candy Dang,[a] Anh N. Hong,[b] Pingyun Feng,*[,b] Xianhui Bu,*[,a]

[a] Department of Chemistry and Biochemistry, California State University, Long Beach, California 90840, United States

[b] Department of Chemistry, University of California, Riverside, California 92521, United States

EXPERIMENTAL SECTION

In this work, for each structural module, we have selected the following: five types of metal trimers (Mg_3, Co_3, Ni_3, Co_2V, and Ni_2V), two L1 ligands (cdc, bcp), and two L2 ligands (tpt, tppy). There are 20 target combinations, ignoring possible variations in M^{2+}/M^{3+} ratios and difference in extra-framework charge-balancing ions. Below, we present the synthesis of 9 combinations (2 from cdc-tpt, 1 from cdc-tppy, 5 from bcp-tpt, and 1 from bcp-tppy), together with a procedure to optimize the synthesis of CPM-33a (Ni3-bdc-tpt).

1. The cdc-tpt (cubane L1-triazine L2) based homo- and hetero-metallic system

Synthesis of CPM-125a-Ni [$Ni_3OH(cdc)_3tpt$][$(CH_3)_2NH_2$] (pacs-Ni3-cdc-tpt, CCDC No. 2159094). In a 15 mL glass vial, 47 mg of nickel(II) chloride hexahydrate ($NiCl_2{\cdot}6H_2O$, ~0.2 mmol), 38 mg cubane-1,4-dicarboxylic acid (H_2cdc, ~0.2 mmol), and 21 mg 2,4,6-tri(4-pyridyl)-1,3,5-triazine (tpt, ~0.67 mmol) were dissolved in 2.5 g dimethylacetamide (DMA) and 0.5 g water. After being stirred for an hour, the vial was placed in a 120 ℃ oven for 3 days, and the mixture was then cooled to room temperature. Green polyhedral crystals were obtained. The phase purity was supported by powder X-ray diffraction.

Synthesis of CPM-125a-CoV [$Co_{1.7}V_{1.3}(OH/O)(bcp)_3tpt$] (pacs-CoV-bcp-tpt). In a 15 mL glass vial, 60 mg of cobalt(II) nitrate hexahydrate ($Co(NO_3)_2{\cdot}6H_2O$, ~0.2 mmol), 16 mg vanadium (Ⅲ) chloride (VCl_3, ~0.2 mmol), 57 mg cubane-1,4-dicarboxylic acid (H_2cdc, ~0.3 mmol), and 31 mg 2,4,6-tri(4-pyridyl)-1,3,5-triazine (tpt, ~0.1 mmol) were dissolved in 6.0 g N-methylformamide (NMF) with 1 drop of concentrated hydrochloric acid. After being stirred for an hour, the vial was placed in a 130 ℃ oven for 3 days, and the mixture was then cooled to room temperature. Dark-red micro crystals were obtained. The phase purity was supported by powder X-ray diffraction.

2. The cdc-tppy (cubane L1-pyridine L2) based homometallic system

Synthesis of CPM-125b-Ni [$Ni_3OH(cdc)_3tppy$][$(CH_3)_2NH_2$] (pacs-Ni3-cdc-tppy). In a 15 mL glass vial, 47 mg of nickel(II) chloride hexahydrate ($NiCl_2 \cdot 6H_2O$, ~0.2 mmol), 38 mg cubane-1,4-dicarboxylic acid (H_2cdc, ~0.2 mmol), and 21 mg 2,4,6-tri(4-pyridyl)pyridine (tppy, ~0.67 mmol) were dissolved in 2.5 g dimethylacetamide (DMA) and 0.5 g water. After being stirred for an hour, the vial was placed in a 120 ℃ oven for 3 days, and the mixture was then cooled to room temperature. Green polyhedral crystals were obtained. The phase purity was supported by powder X-ray diffraction.

3. The bcp-tpt based homo- and hetero-metallic System

Synthesis of CPM-111a-Ni [$Ni_3OH(bcp)_3tpt$][$(CH_3)_2NH_2$] (pacs-Ni3-bcp-tpt, CCDC No. 2159092). In a 15 mL glass vial, 47 mg of nickel(II) chloride hexahydrate ($NiCl_2 \cdot 6H_2O$, ~0.2 mmol), 31 mg bicyclo[1.1.1]pentane-1,3-dicarboxylic acid (H_2bcp, ~0.2 mmol), and 21 mg 2,4,6-tri(4-pyridyl)-1,3,5-triazine (tpt, ~0.67 mmol) were dissolved in 2.5 g dimethylacetamide (DMA) and 0.5 g water. After being stirred for an hour, the vial was placed in a 120 ℃ oven for 3 days, and the mixture was then cooled to room temperature. Green polyhedral crystals were obtained. The phase purity was supported by powder X-ray diffraction.

Synthesis of CPM-111a-CoV [$Co_{1.6}V_{1.4}(O/OH)(bcp)_3tpt$] (pacs-CoV-bcp-tpt). In a 15 mL glass vial, 120 mg of cobalt(II) nitrate hexahydrate ($Co(NO_3)_2 \cdot 6H_2O$, ~0.4 mmol), 32 mg vanadium (Ⅲ) chloride (VCl_3, ~0.2 mmol), 94 mg bicyclo[1.1.1]pentane-1,3-dicarboxylic acid (H_2bcp, ~0.6 mmol), and 62 mg 2,4,6-tri(4-pyridyl)-1,3,5-triazine (tpt, ~0.2 mmol) were dissolved in 3.0 g dimethylformamide (DMF). After being stirred for an hour, the vial was placed in a 130 ℃ oven for 3 days, and the mixture was then cooled to room temperature. Dark-red micro crystals were obtained. The phase purity was supported by powder X-ray diffraction.

Synthesis of CPM-111a-NiV [$Ni_{1.9}V_{1.1}OH(bcp)_3tpt$] (pacs-NiV-bcp-tpt). In a 15 mL glass vial, 116 mg of nickle(II) nitrate hexahydrate ($Ni(NO_3)_2 \cdot 6H_2O$, ~0.4 mmol), 32 mg vanadium (Ⅲ) chloride (VCl_3, ~0.2 mmol), 94 mg bicyclo[1.1.1]pentane-1,3-dicarboxylic acid (H_2bcp, ~0.6 mmol), and 62 mg 2,4,6-tri(4-pyridyl)-1,3,5-triazine (tpt, ~0.2 mmol) were dissolved in 3.0 g dimethylformamide (DMA) and 0.5 g of H_2O with the addition of 4 drops of concentrated hydrochloric acid. After being stirred for an hour, the vial was placed in a 130 ℃ oven for 3 days, and the mixture was then cooled to room temperature. Yellow micro crystals were obtained. The phase purity was supported by powder X-ray diffraction.

Synthesis of CPM-111a-Co [$Co_3(bcp)_3tpt(CH_3)_2NH_2$]] (pacs-Co3-bcp-tpt, CCDC No. 2159091). In a 15 mL glass vial, 143mg of cobalt(II) chloride hexahydrate ($CoCl_2 \cdot 6H_2O$, ~0.6 mmol), 94 mg bicyclo[1.1.1]pentane-1,3-dicarboxylic acid (H_2bcp, ~0.6 mmol), and 62 mg 2,4,6-tri(4-pyridyl)-1,3,5-triazine (tpt, ~0.2 mmol) were dissolved in 4.0 g dimethylacetamide (DMA) and 2 ml 1,3-dimethyl 3,4,5,6-tetrahydro-2(1H)-pyrimidinone (DMPU) with the addition of 28 μL 1,1,1,5,5,5-hexafluoro-2,4- pentanedione (HFP). After being stirred for an hour, the vial was placed in a 130 ℃ oven for 4 days, and the mixture was then cooled to room temperature. Red polyhedral crystals were obtained. The phase purity was supported by powder X-ray diffraction.

Synthesis of CPM-111a-Mg [$Mg_3(bcp)_3tpt(CH_3)_2NH_2$]] (pacs-Mg3-bcp-tpt, CCDC No. 2159093). In a 15 mL glass vial, 75 mg $Mg(NO_3)_2 \cdot 6H_2O$ (0.3 mmol), 47 mg bicyclo[1.1.1]pentane-1,3-dicarboxylic acid (H_2bcp, ~0.3 mmol), and 31 mg 2,4,6-tri(4-pyridyl)-1,3,5-triazine (tpt, ~0.1 mmol) were dissolved in 4.0 g dimethylacetamide (DMA) and 2 ml 1,3-dimethyl 3,4,5,6-tetrahydro-2(1H)-pyrimidinone (DMPU) with the addition of 28 μL 1,1,1,5,5,5-hexafluoro-2,4- pentanedione (HFP). After being stirred for an hour, the vial was placed in a 120 ℃ oven for 4 days, and the mixture was then cooled to room temperature. Yellowish polyhdral crystals were obtained. Alternatively, CPM-111a-Mg can be synthesized in a similar condition except that the solvent of DMA and DMPU was replaced by 6 mL N-methylformamide (NMF). The

samples synthesized in NMF was used for gas adsorption measurements. The phase purity was supported by powder X-ray diffraction.

4. The bcp-tppy based system

Synthesis of CPM-111b-Ni [$Ni_3OH(bcp)_3tppy(CH_3)_2NH_2$]] (pacs-Ni3-bcp-tppy). In a 15 mL glass vial, 47 mg of nickel(II) chloride hexahydrate ($NiCl_2{\cdot}6H_2O$, ~0.2 mmol), 31 mg bicyclo[1.1.1]pentane-1,3-dicarboxylic acid (H_2bcp, ~0.2 mmol), and 21 mg 2,4,6-tri(4-pyridyl)pyridine (tppy, ~0.67 mmol) were dissolved in 2.5 g dimethylacetamide (DMA) and 0.5 g water with addition of 1 drop of concentrated hydrochloric acid. After being stirred for an hour, the vial was placed in a 120 ℃ oven for 3 days, and the mixture was then cooled to room temperature. Green polyhedral crystals were obtained. The phase purity was supported by powder X-ray diffraction.

5. The bdc-tpt based system

Synthesis of CPM-33a [$Ni_3OH(bdc)_3tpt(CH_3)_2NH_2$]] (pacs-Ni3-bdc-tpt). CPM-33a was repeated using a modified synthetic procedure.[S1] In a 15 mL glass vial, 87 mg of nickel nitrate hexahydrate ($Ni(NO_3)_2{\cdot}6H_2O$, ~0.3 mmol), 50 mg terephthalic acid (H_2bdc, ~0.3 mmol), and 31 mg 2,4,6-tri(4-pyridyl)-1,3,5-triazine (tpt, ~0.1 mmol) were dissolved in 4.0 g dimethylformamide (DMF) and 0.5 g water with the addition of 80 μL concentrated HCl. After being stirred for an hour, the vial was placed in a 130 ℃ oven for 5 days, and the mixture was then cooled to room temperature. Green polyhedral crystals were obtained. The phase purity was supported by powder X-ray diffraction.

Single-Crystal X-ray Diffraction Characterization. The single-crystal X-ray diffraction measurements were performed on a Bruker diffractometer using graphite-monochromated MoKα (λ= 0.71073 Å) radiation

at room temperature. Diffraction data were integrated and scaled by 'multi-scan' method with the Bruker APEX software. The structure was solved by intrinsic phasing which was embedded in 'APEX Ⅲ' software and the refinement against all reflections of the compound was performed using 'APEX Ⅲ'. All non-hydrogen framework atoms were refined anisotropically. All the hydrogen atoms were calculated. CCDC 2159091-2159094 contain the supplementary crystallographic data for this paper. These data are provided free of charge by the Cambridge Crystallographic Data Centre.

Powder X-ray Diffraction (PXRD) Characterization. Powder X-ray diffraction experiments were performed on a PANalytical X'Pert Pro MPD diffractometer, equipped with a linear X'Celerator detector, which was operating at 40 kV and 35 mA (Cu Kα radiation, λ = 1.5418 Å). The data collection was performed at room temperature in the range from 5° to 40° with a step size of ~0.008°. The simulated powder pattern was obtained from the single crystal data.

Thermogravimetric (TG) Measurement. A TA Instruments TGA Q500 thermal analyzer was used to measure the TG curve by heating the sample from 30 ℃ to 800 ℃ with heating rate of 5℃/min under nitrogen flow. The flow rate of the nitrogen gas was controlled at about 60 milliliters per minute.

^{1}H NMR Spectra. A Bruker Avance DPX-300 NMR Spectrometer (400 MHz) was used to test ^{1}H NMR spectra. The relaxation delay (d1) was set to 20 seconds to ensure that reliable integrals were obtained, allowing for the relative concentrations of the molecular components to be accurately determined.

Water Stability Tests for CPM-111a-Ni (Ni3-bcp-tpt) and CPM-33 (Ni3-bdc-tpt). To avoid the influence from different synthetic batches on gas uptake, samples after gas adsorptions were used to test water stability. Around 120 mg post-adsorption sample were put in 40 mL water. After 24 hours, the samples were washed by ethanol and dried in vacuum oven, which were used for PXRD and gas adsorption experiments.

Energy dispersive spectroscopy (EDS). The semi-quantitative elemental analyses of heterometallic MOF samples were performed by using a TESCAN Vega3 SBH emission scanning electron microscope equipped with 30 mm^2 SD energy dispersive spectroscopy (EDS) detector. Data acquisition was performed with an accelerating voltage of 15-20 kV and 20 s accumulation time.

Gas Sorption Measurement. Gas sorption measurements were carried out on a Micromeritics ASAP 2020 and ASAP 2020 PLUS Physisorption Analyzers. Prior to the measurement, the as-synthesized sample was purified by DMF and immersed in methanol (extra dry) for three days. During each day, the solution was refreshed. After solvent exchange, the upper solvent was decanted. The sample was first dried under air flow gently and was subsequently transferred into the test tube. The degas process was performed under room temperature for 2 hours and further dried at 60 ^{o}C for another 10 hours.

Isosteric Heat of Adsorption (Q_{st}). The isosteric heats of adsorption for all the gases were calculated using the isotherms at 273 K and 298 K, following the Clausius-Clapeyron equation. It was done with the calculation program embedded in the software of ASAP 2020 plus. High accuracy of the Q_{st} was found in all the calculations as evidenced by the linearity in the isosters.

Selectivity by IAST. To evaluate the C_2H_2/CO_2 separation performance, the selectivity was calculated by ideal adsorbed solution theory (IAST). Dual-Site Langmuir-Freundlich (DSLF) model was employed to fit the gas adsorption isotherms over the entire pressure range. DSLF model can be written as:

$$N = \frac{A_1 B_1 p^{1/n_1}}{1 + B_1 p^{1/n_1}} + \frac{A_2 B_2 p^{1/n_2}}{1 + B_2 p^{1/n_2}} \tag{1}$$

Where N is the quantity adsorbed, p is the pressure of bulk gas at equilibrium with adsorbed phase, A_i is the saturation loadings for adsorption site i (i=1 or 2), and B_i are the affinity parameters. $1/n_i$ is the index of heterogeneity. The R factors for all the fitting are close to or higher than 99.999%.

The detailed methodology for calculating the amount of A and B adsorption from a mixture by IAST is described elsewhere.[S2] The adsorption selectivity is finally defined as:

$$selectivity = \frac{q_A / q_B}{p_A / p_B} \tag{2}$$

where q_i (i = A or B) is the uptake quantity in the mixture and p_i is the feeding partial pressure of component i.

Separation Potential.[S3] The separation potential (ΔQ) is a combined metric, which considering both uptake capacity and selectivity. It is defined to quantify mixture separations in fixed bed adsorbers. For a C_2H_2/CO_2 mixture with mole fractions y_{C2H2}, and y_{CO2}=1-y_{C2H2}, the gravimetric separation potential ΔQ, is calculated from IAST using the formula

$$\Delta Q = q_{C_2H_2} \frac{y_{CO_2}}{1-y_{CO_2}} - q_{CO_2} \tag{3}$$

where q_{C2H2} and q_{CO2} are C_2H_2 and CO_2 uptake in the mixture, respectively, which are calculated based on IAST theory. For 50/50 mixture, the formula (3) can be simplified as

$$\Delta Q = q_{C_2H_2} - q_{CO_2} \tag{4}$$

The physical significance of ΔQ is that it represents the maximum amount of pure C_2H_2 that can be recovered during the adsorption phase of fixed bed separations.

Breakthrough Experiments. The breakthrough experiments were performed by a homemade breakthrough experimental setup. The crystals of Ni3-bcp-tpt were synthesized and were immediately subject to solvent exchange in methanol for 3 days. After solvent exchange, the samples were activated in a vacuum oven at room temperature overnight and were further degassed on Micromeritics ASAP2020 gas adsorption instrument at 80 degree for three days. The activated samples are pelletized and broken into fragments.

Samples with size between 250 to 354 micron were selected by sieves and collected. 1.598 g samples were loaded into a U shape glass tube. A C_2H_2/CO_2/He mixture (1/1/2 mL/min) flowed through the U shape tube with effluent gas monitored by Shimdazu GC-2030 gas chromatography with FID and TCD detectors. Samples are regenerated by heating at 80 ^{o}C under 10 mL/min He overnight.

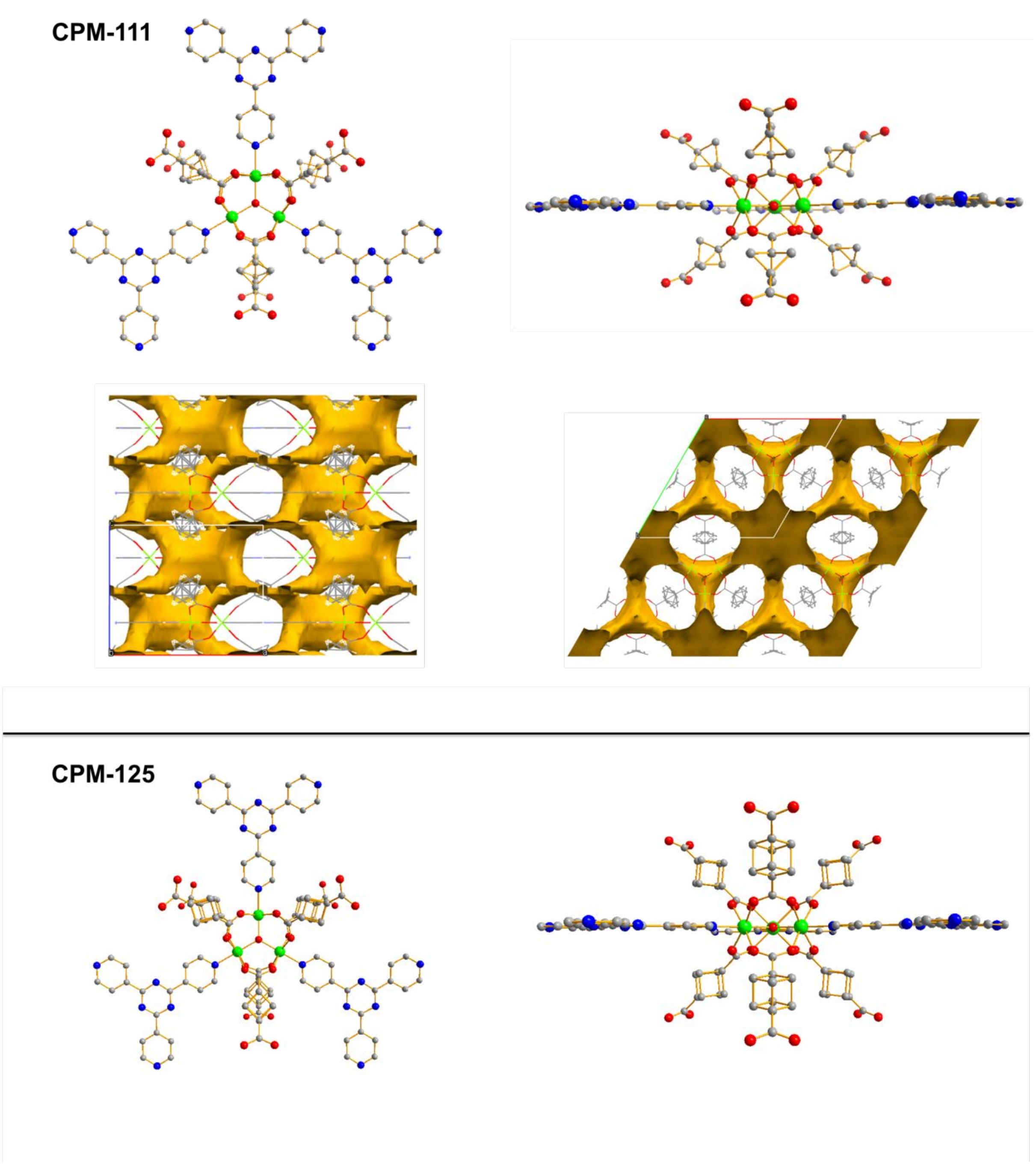

Figure S1. Trimer building blocks in bcp-based CPM-111 series and cdc-based CPM-125 series projected down the c and a axes, respectively. Metal: green; O: red; N: blue; Carbon: gray. The hydrogen atoms are deleted for clarity. The pore surface for CPM-111 series viewed along a axis (left) and c axis (right) is also shown.

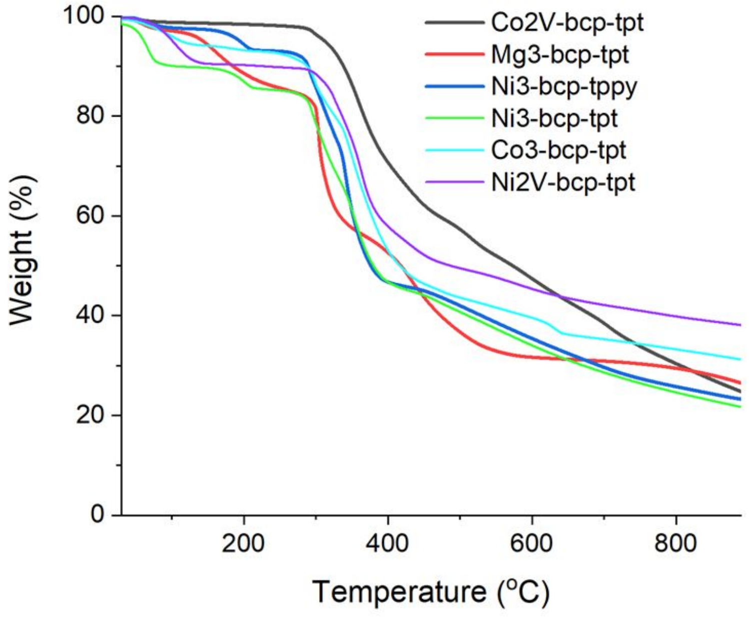

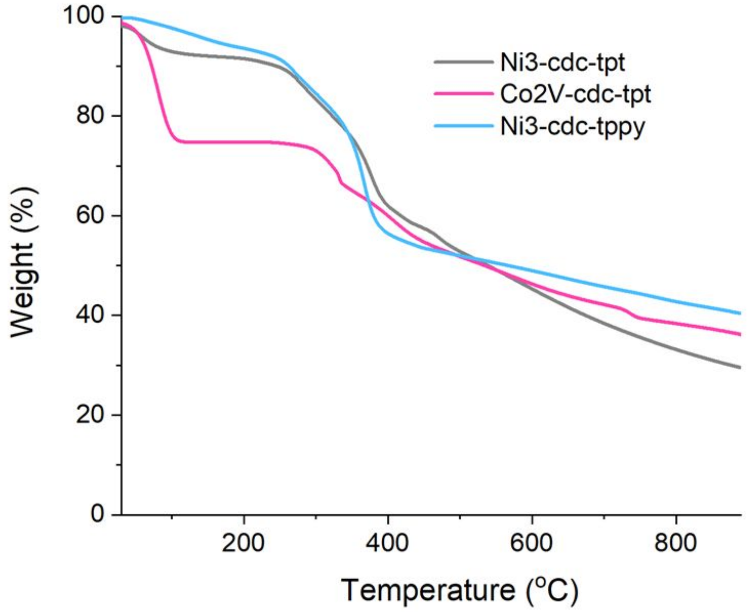

Figure S2. TGA data of solvent-exchanged bcp-based pacs (CPM-111 series) and cdc-based pacs (CPM-125 series) reported in this work.

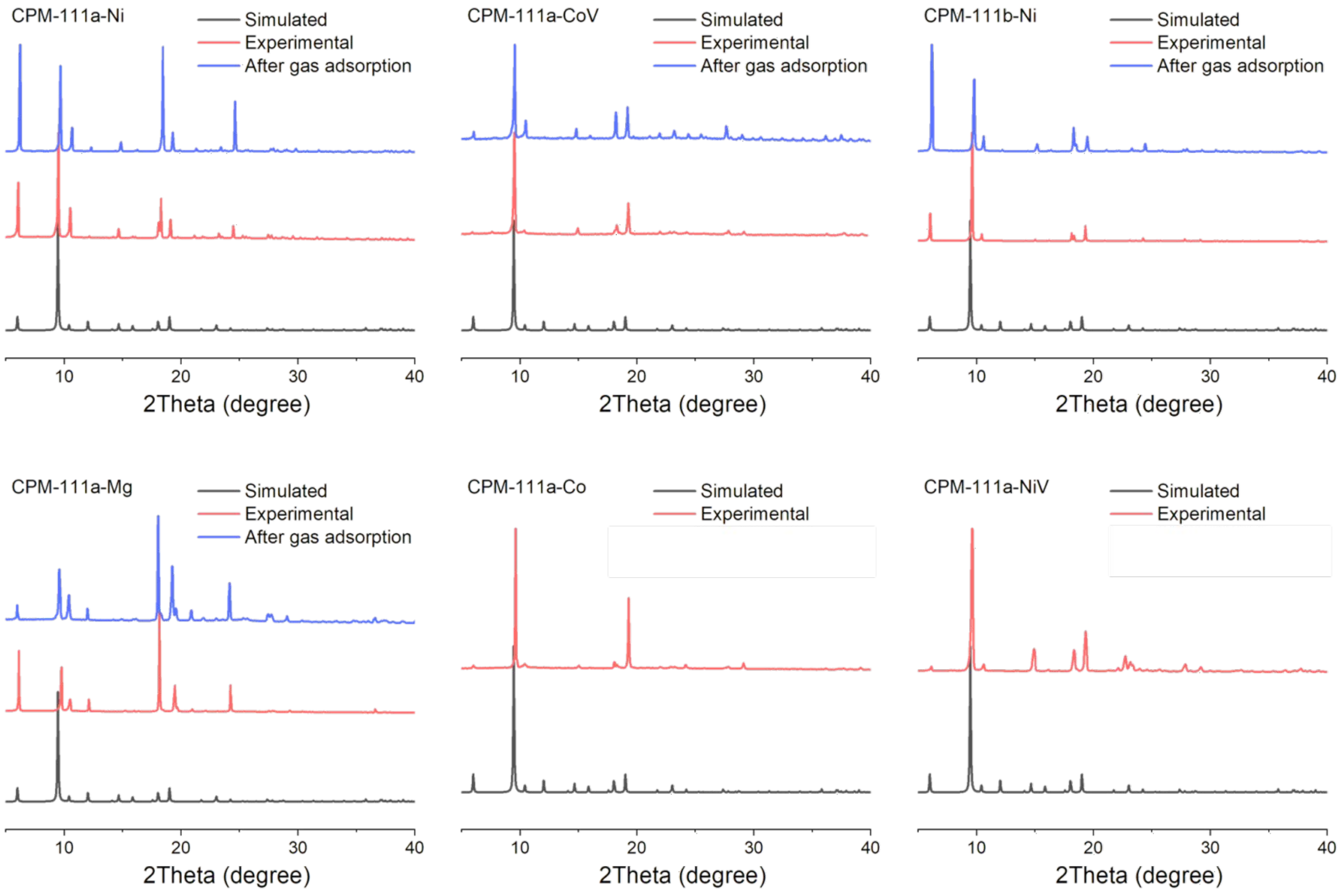

CPM-111a-Ni
Simulated
Experimental
After gas adsorption
10
20
30
40
2Theta (degree)
CPM-111a-CoV
Simulated
Experimental
After gas adsorption
10
20
30
40
2Theta (degree)
CPM-111b-Ni
Simulated
Experimental
After gas adsorption
10
20
30
40
2Theta (degree)
CPM-111a-Mg
Simulated
Experimental
After gas adsorption
10
20
30
40
2Theta (degree)
CPM-111a-Co
Simulated
Experimental
10
20
30
40
2Theta (degree)
CPM-111a-NiV
Simulated
Experimental
10
20
30
40
2Theta (degree)

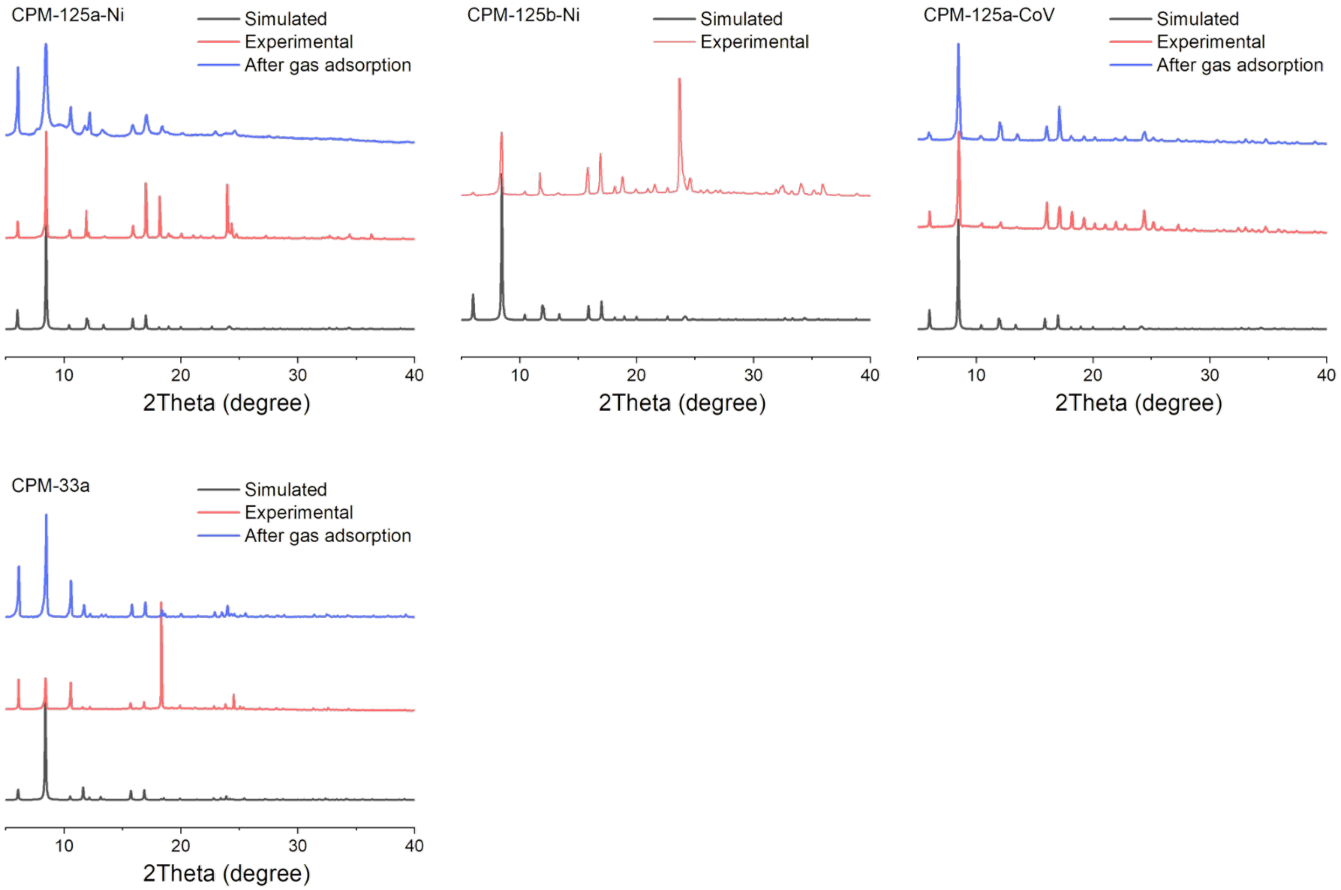

Figure S3. Simulated, Experimental PXRD patterns for all the MOFs reported in this work. PXRD pattern of samples after gas adsorption is also shown, which demonstrates their robust nature. The simulated pattern for CPM-111 series and CPM-125 series are generated from CPM-111a-Ni and CPM-125a-Ni, respectively.

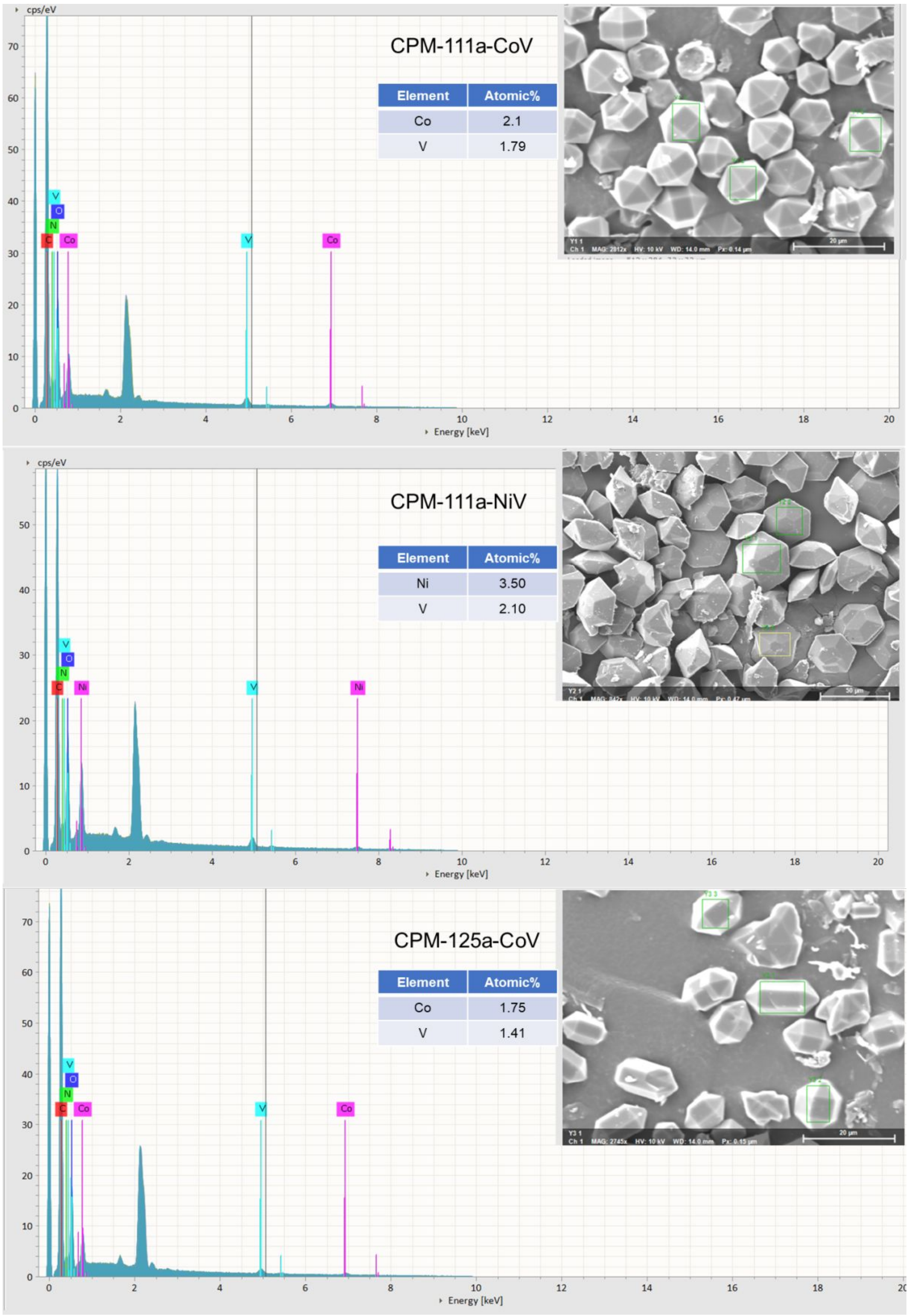

Figure S4. EDS spectra of heterometallic MOFs reported in this work. The atomic ratios are averaged by three measurements from different crystals.

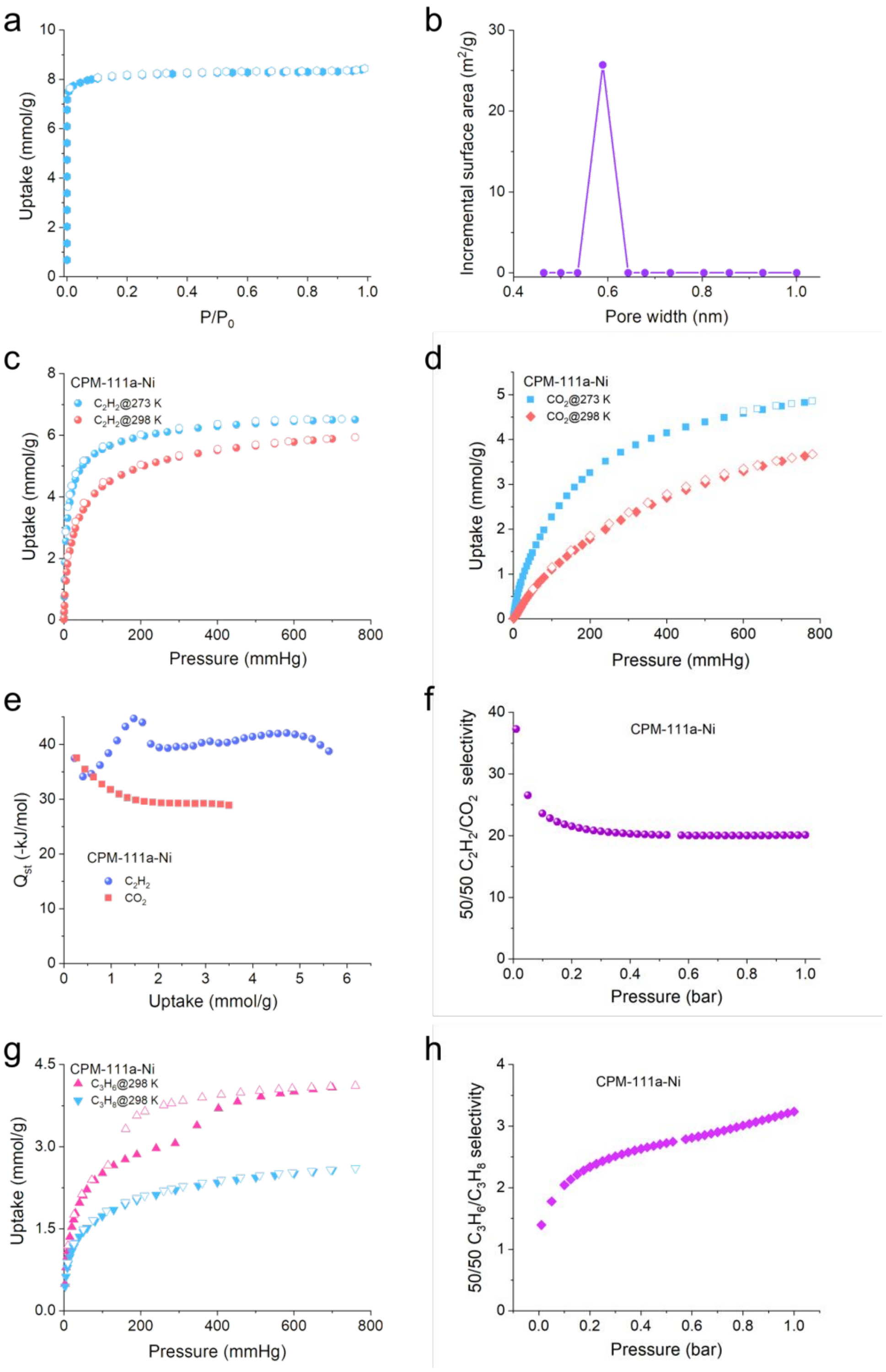

Figure S5. Gas adsorption properties of CPM-111a-Ni. a: N_2 adsorption isotherm at 77 K; b: DFT pore size distribution; c: C_2H_2 adsorption isotherms at 273 K and 298 K; d: CO_2 adsorption isotherms at 273 K and 298 K; e: Adsorption enthalpy (Q_{st}) for C_2H_2 and CO_2; f: IAST selectivity for 50/50 C_2H_2/CO_2. g: C_3H_6 and C_3H_8 adsorption isotherms at 298 K. h. IAST selectivity for 50/50 C_3H_6/C_3H_8. The pressure range of $P/P_0 < 10^{-3}$ was applied to calculate the pore size due to the micro-porous feature of this family of materials.

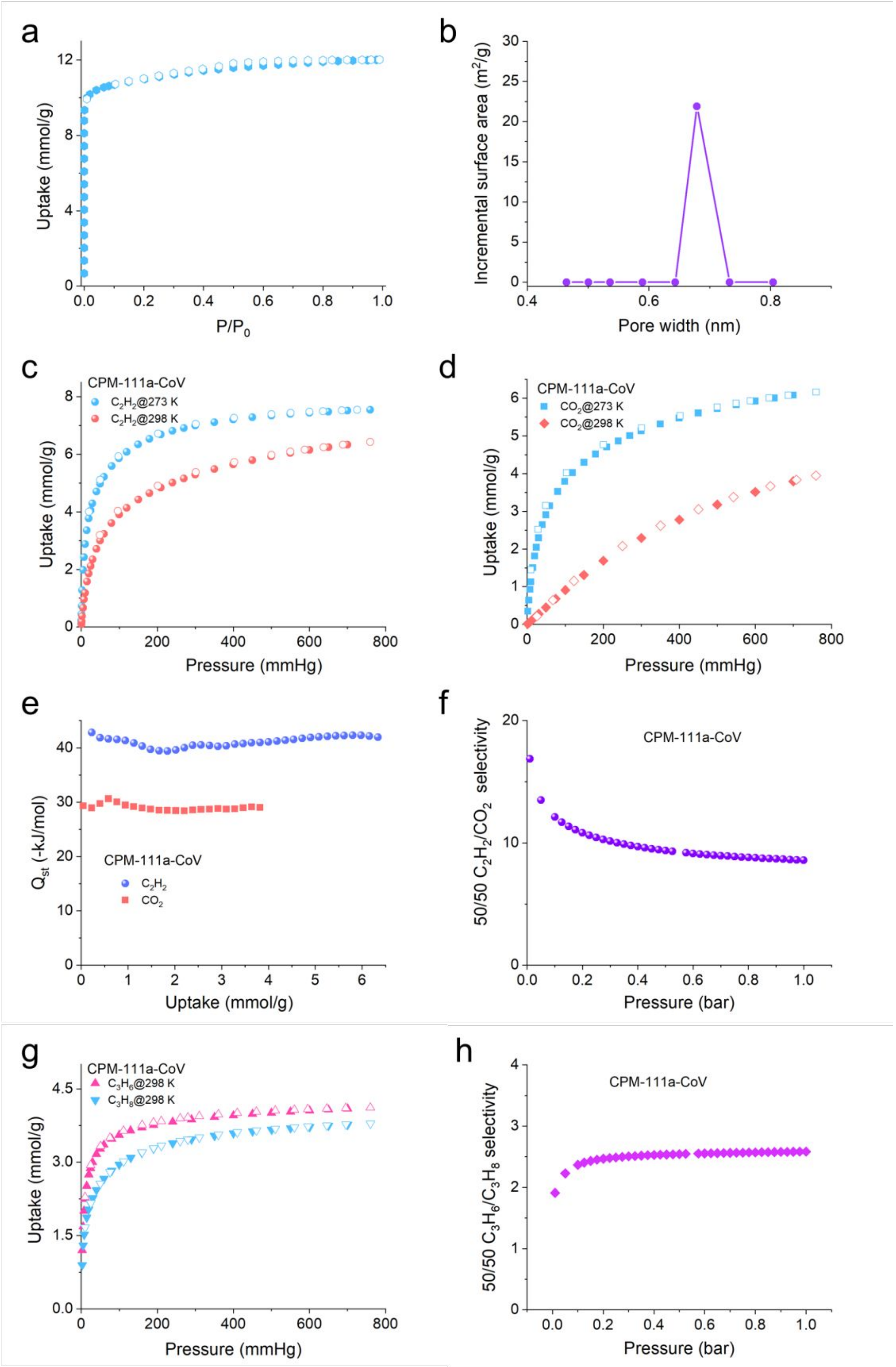

Figure S6. Gas adsorption properties of CPM-111a-CoV. a: N_2 adsorption isotherm at 77 K; b: DFT pore size distribution; c: C_2H_2 adsorption isotherms at 273 K and 298 K; d: CO_2 adsorption isotherms at 273 K and 298 K; e: Adsorption enthalpy (Q_{st}) for C_2H_2 and CO_2; f: IAST selectivity for 50/50 C_2H_2/CO_2. g: C_3H_6 and C_3H_8 adsorption isotherms at 298 K. h. IAST selectivity for 50/50 C_3H_6/C_3H_8. The pressure range of $P/P_0 < 10^{-3}$ was applied to calculate the pore size due to the micro-porous feature of this family of materials.

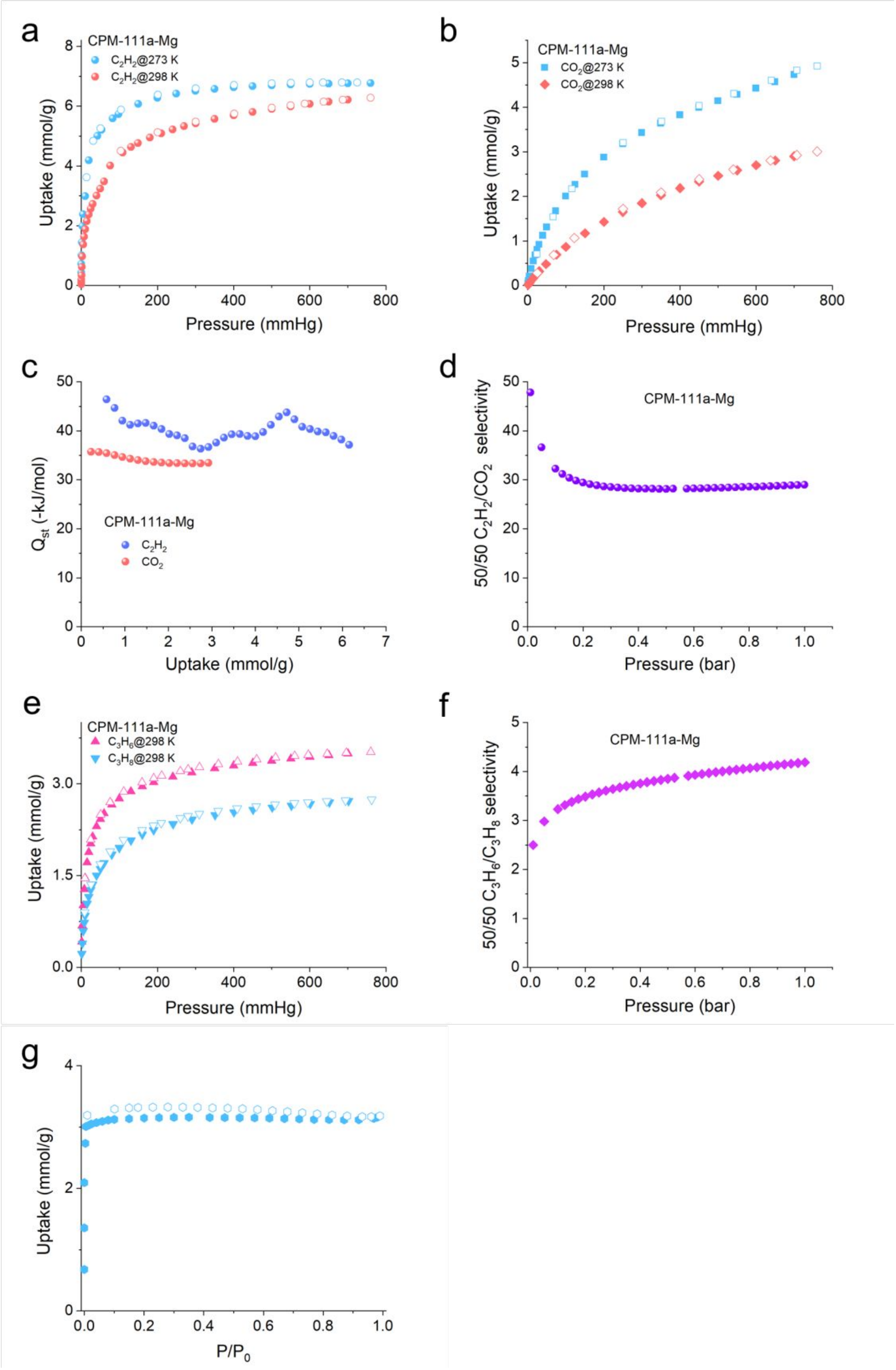

Figure S7. Gas adsorption properties of CPM-111a-Mg. a: C_2H_2 adsorption isotherms at 273 K and 298 K; b: CO_2 adsorption isotherms at 273 K and 298 K; c: Adsorption enthalpy (Q_{st}) for C_2H_2 and CO_2; d: IAST selectivity for 50/50 C_2H_2/CO_2; e: C3H6 and C3H8 adsorption isotherms at 298 K. f. IAST selectivity for 50/50 C3H6/C3H8. g: N_2 adsorption isotherm at 77 K;.

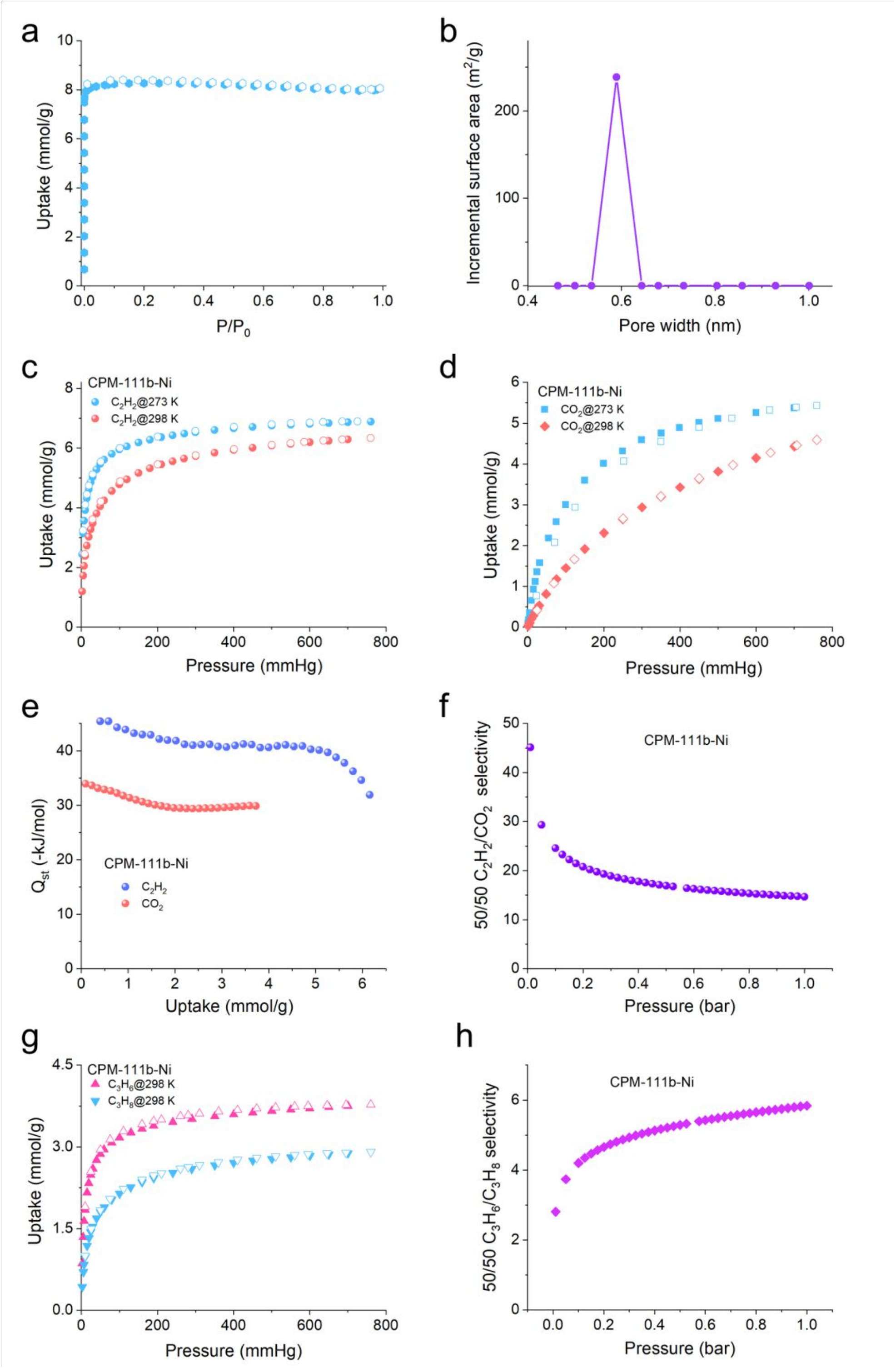

Figure S8. Gas adsorption properties of CPM-111b-Ni. a: N_2 adsorption isotherm at 77 K; b: DFT pore size distribution; c: C_2H_2 adsorption isotherms at 273 K and 298 K; d: CO_2 adsorption isotherms at 273 K and 298 K; e: Adsorption enthalpy (Q_{st}) for C_2H_2 and CO_2; f: IAST selectivity for 50/50 C_2H_2/CO_2. g: C_3H_6 and C_3H_8 adsorption isotherms at 298 K. h. IAST selectivity for 50/50 C_3H_6/C_3H_8. The pressure range of $P/P_0 < 10^{-3}$ was applied to calculate the pore size due to the micro-porous feature of this family of materials.

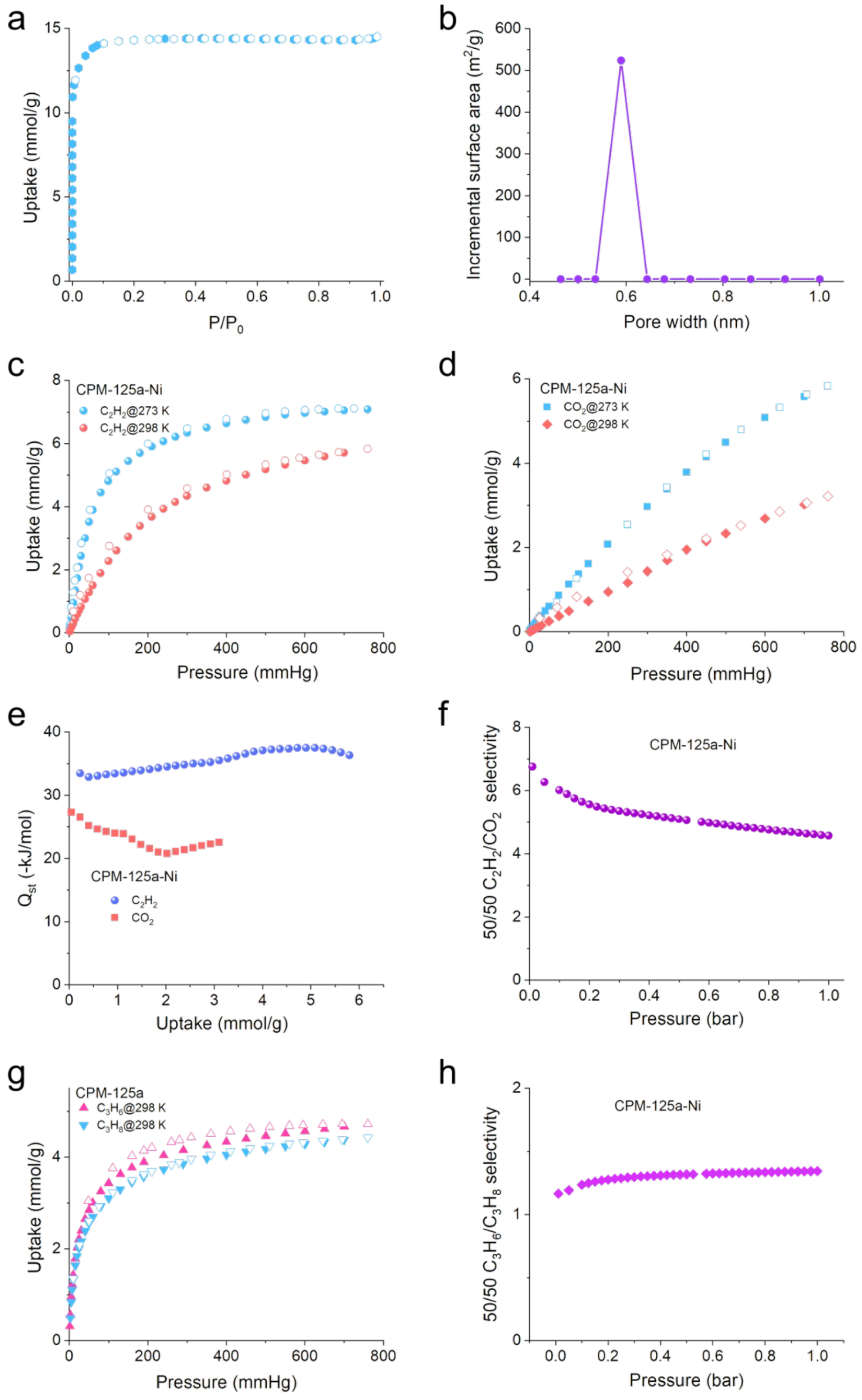

Figure S9. Gas adsorption properties of CPM-125a-Ni. a: N_2 adsorption isotherm at 77 K; b: DFT pore size distribution; c: C_2H_2 adsorption isotherms at 273 K and 298 K; d: CO_2 adsorption isotherms at 273 K and 298 K; e: Adsorption enthalpy (Q_{st}) for C_2H_2 and CO_2; f: IAST selectivity for 50/50 C_2H_2/CO_2. g: C_3H_6 and C_3H_8 adsorption isotherms at 298 K. h. IAST selectivity for 50/50 C_3H_6/C_3H_8. The pressure range of $P/P_0 < 10^{-3}$ was applied to calculate the pore size due to the micro-porous feature of this family of materials.

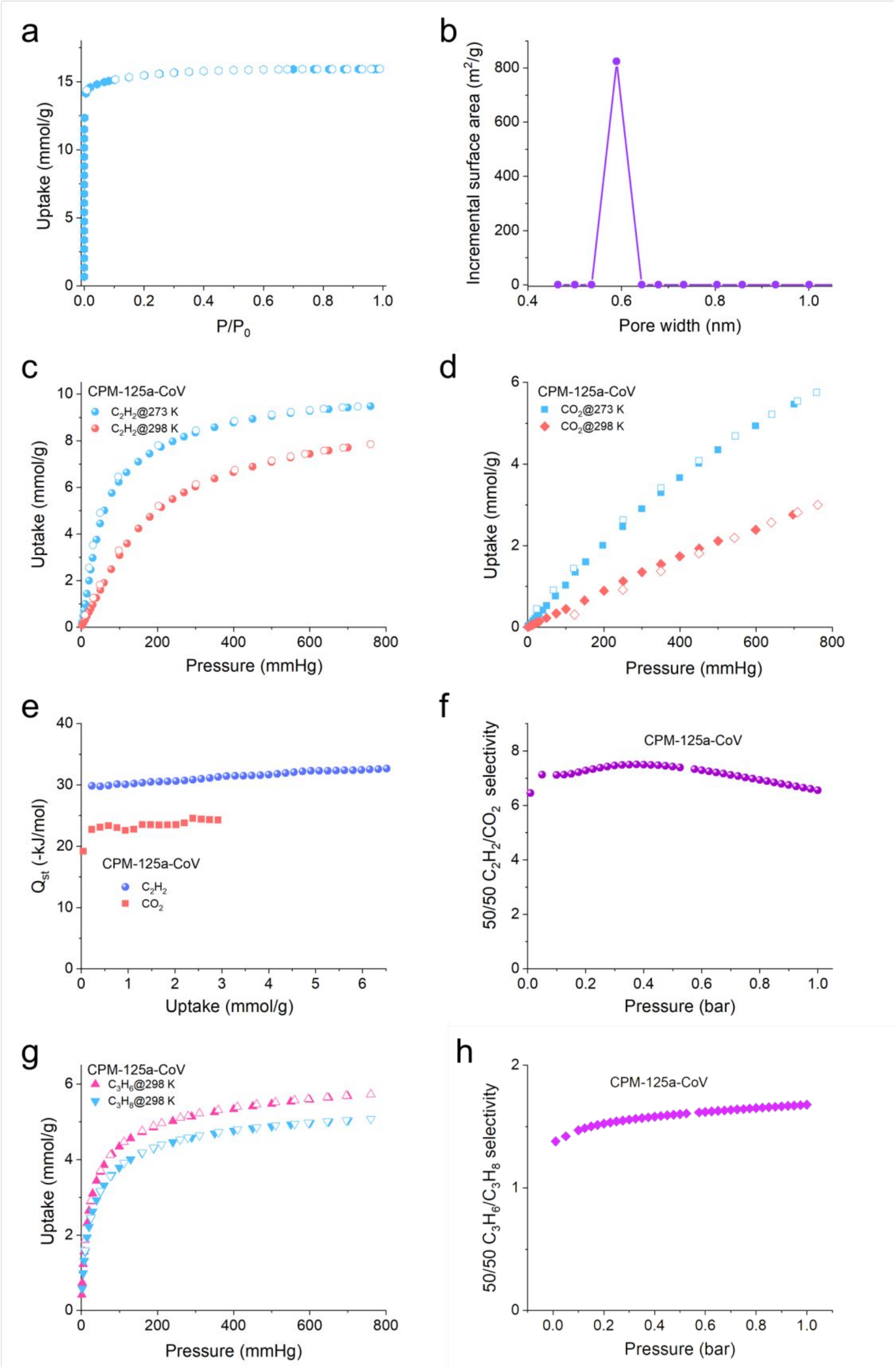

Figure S10. Gas adsorption properties of CPM-125a-CoV. a: N_2 adsorption isotherm at 77 K; b: DFT pore size distribution; c: C_2H_2 adsorption isotherms at 273 K and 298 K; d: CO_2 adsorption isotherms at 273 K and 298 K; e: Adsorption enthalpy (Q_{st}) for C_2H_2 and CO_2; f: IAST selectivity for 50/50 C_2H_2/CO_2. g: C_3H_6 and C_3H_8 adsorption isotherms at 298 K. h. IAST selectivity for 50/50 C_3H_6/C_3H_8. The pressure range of $P/P_0 < 10^{-3}$ was applied to calculate the pore size due to the micro-porous feature of this family of materials.

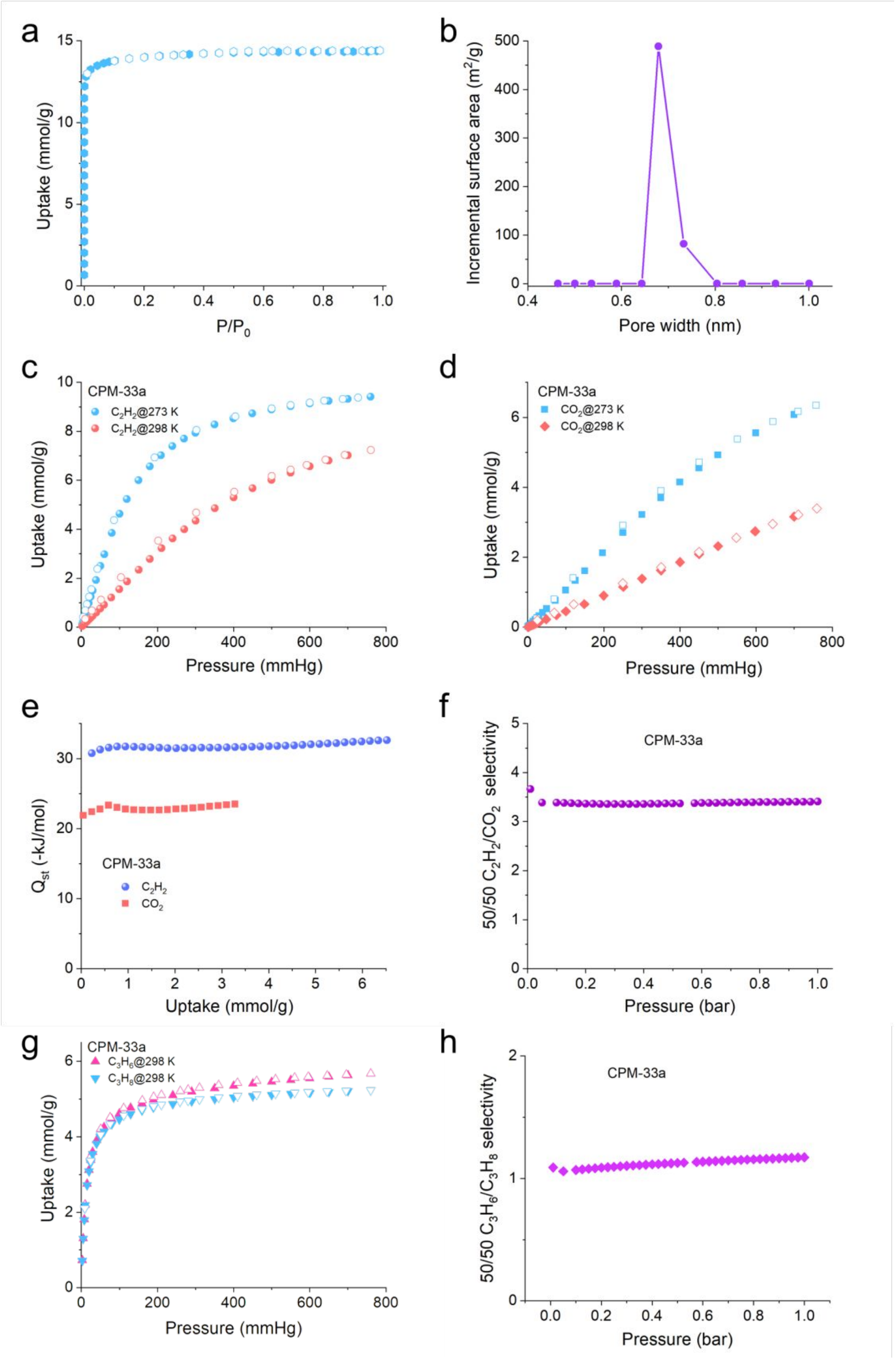

Figure S11. Gas adsorption properties of CPM-33a. a: N_2 adsorption isotherm at 77 K; b: DFT pore size distribution; c: C_2H_2 adsorption isotherms at 273 K and 298 K; d: CO_2 adsorption isotherms at 273 K and 298 K; e: Adsorption enthalpy (Q_{st}) for C_2H_2 and CO_2; f: IAST selectivity for 50/50 C_2H_2/CO_2. g: C_3H_6 and C_3H_8 adsorption isotherms at 298 K. h. IAST selectivity for 50/50 C_3H_6/C_3H_8. The pressure range of $P/P_0 < 10^{-3}$ was applied to calculate the pore size due to the micro-porous feature of this family of materials.

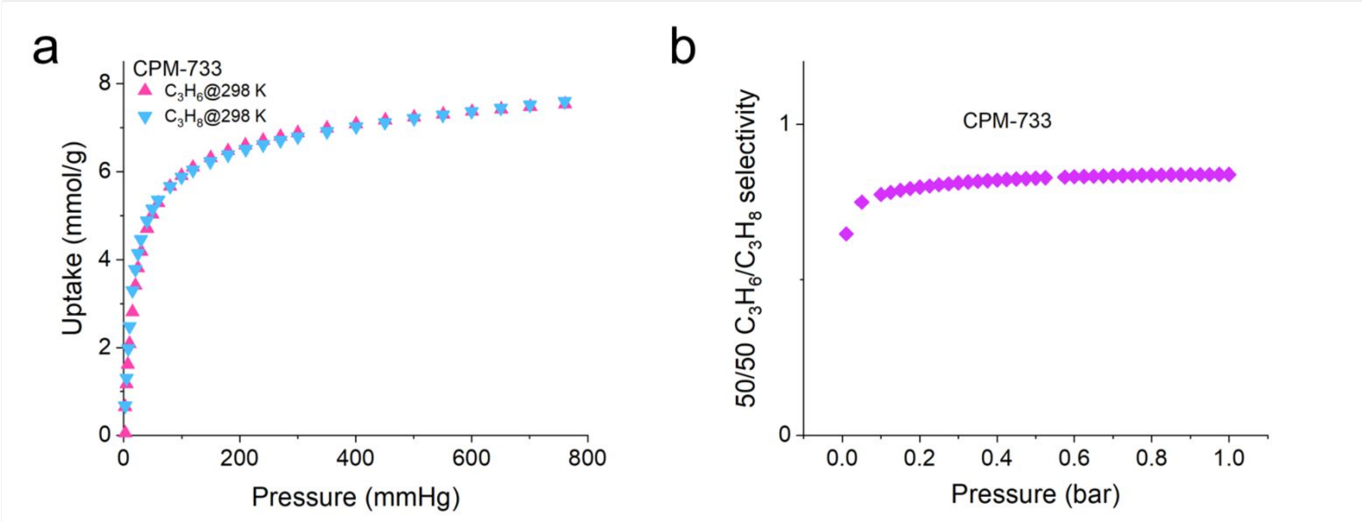

Figure S12. C3 adsorption properties of CPM-733 (Co2V-bdc-tpt). a: C_3H_6 and C_3H_8 adsorption isotherms at 298 K. b. IAST selectivity for 50/50 C_3H_6/C_3H_8. Other gas adsorption isotherms could be accessed from Ref. S4,5.

Chemical Shift	Assignments
9.40, 9.29	-CH in tpt
2.08	-CH2 in bcp ligand
2.44	-CH3 in $[NH_2(CH_3)_2]^+$
6.75	DCl
2.51	DMSO-d_6

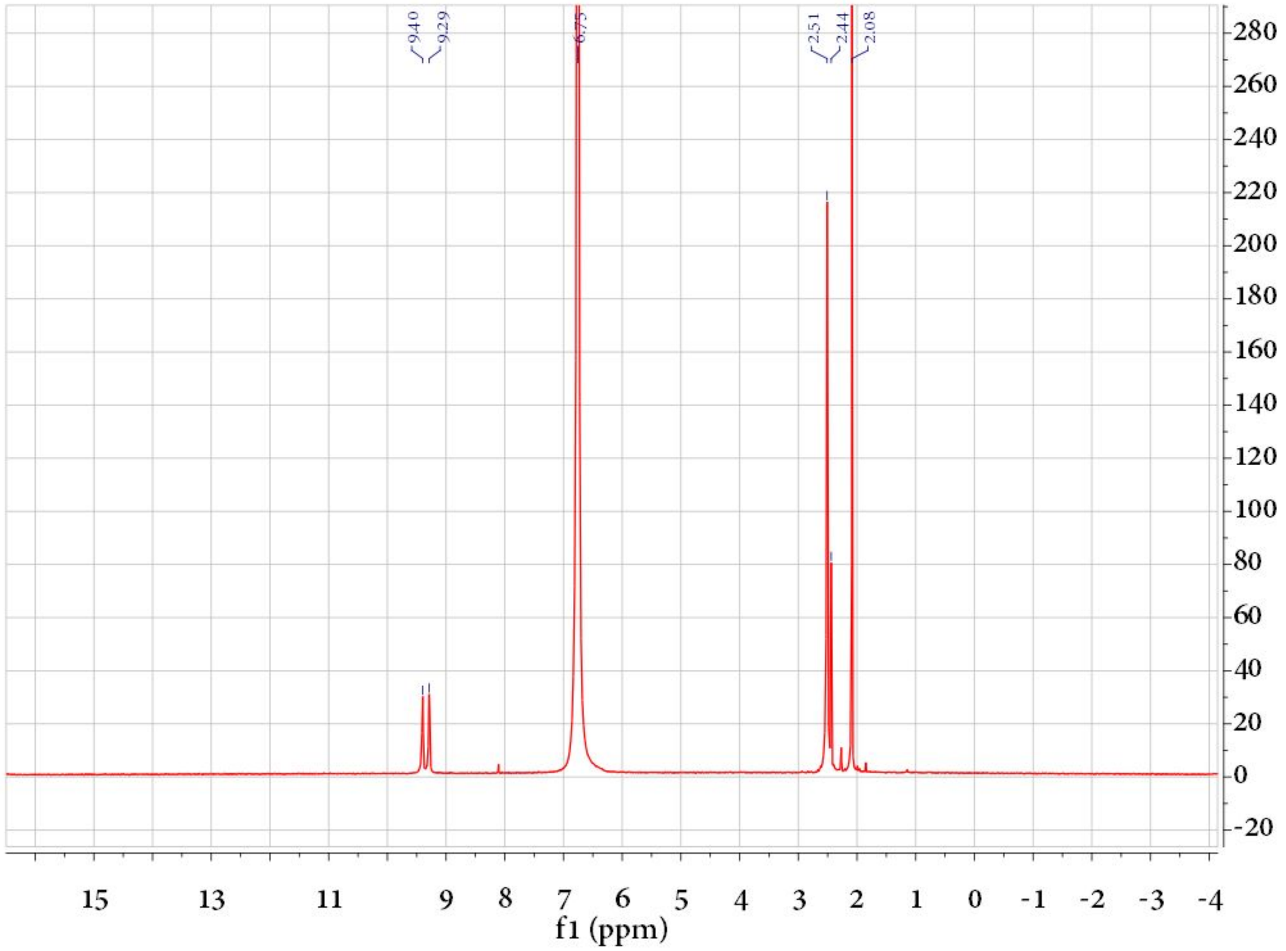

Figure S13. ^{1}H NMR spectrum of activated CPM-111a-Ni digested in a mixture of DMSO-d_6, DCl in D_2O. Minor impurities have been observed and are attributed to residual solvents in the pore. The observation of methyl peaks from $[NH_2(CH_3)_2]^+$ indicates the anionic feature of the framework.

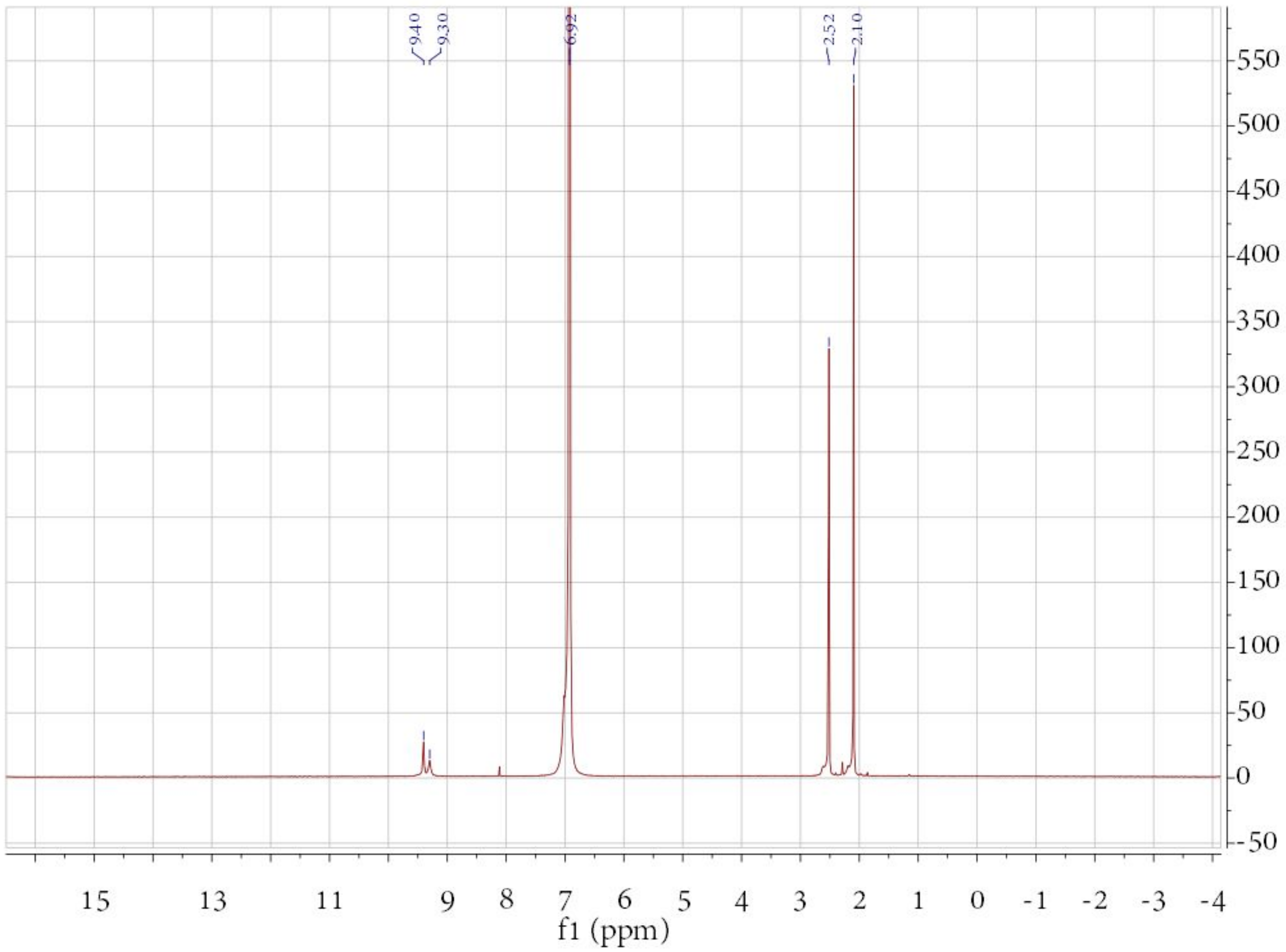

Figure S14. ^{1}H NMR spectrum of activated CPM-111a-CoV digested in a mixture of DMSO-d_6, DCl in D_2O. Minor impurities have been observed and are attributed to residual solvents in the pore. The absence of methyl peaks from $[NH_2(CH_3)_2]^+$ indicates the neutral feature of the framework.

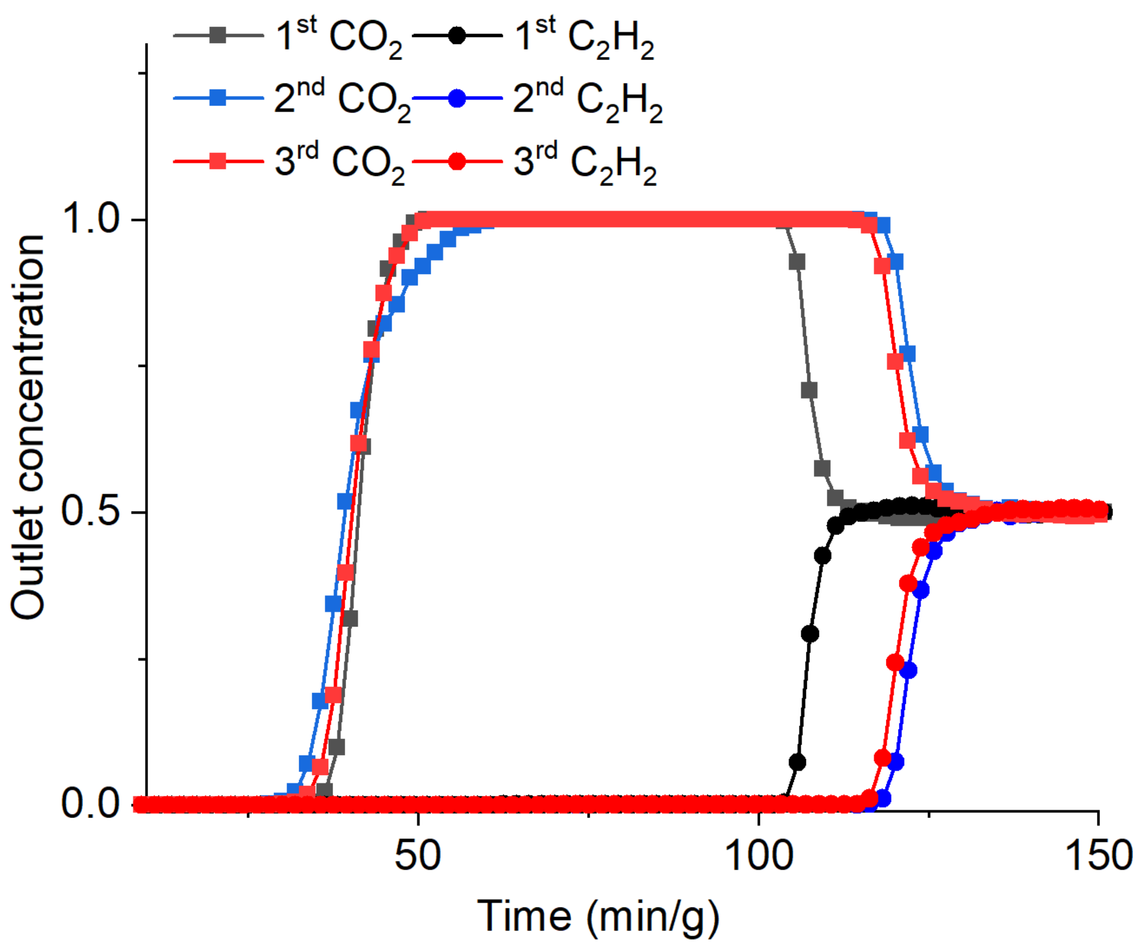

Figure S15. Three cycles of Breakthrough curves at 1 atm and 298 K with a flow of $C_2H_2/CO_2/He$ (1/1/2 mL/min) for Ni3-bcp-tpt. The breakthrough time of the second and third cycles is close and is a little bit longer than the first one, which could be caused by the more complete activation.

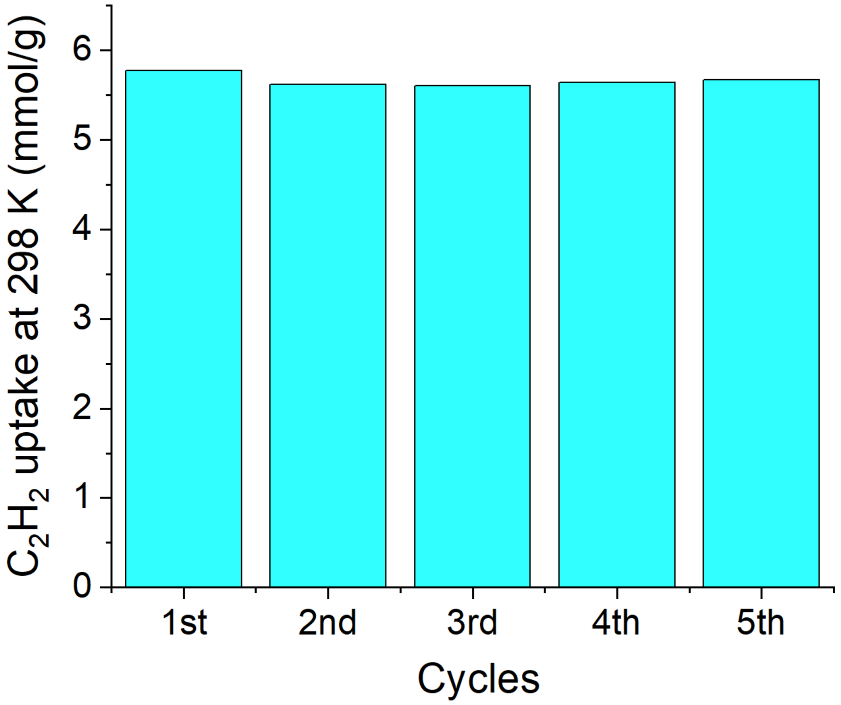

Figure S16. Multiple C_2H_2 adsorption tests of Ni3-bcp-tpt at 298 K, showing almost no loss of capacity during 5 cycles.

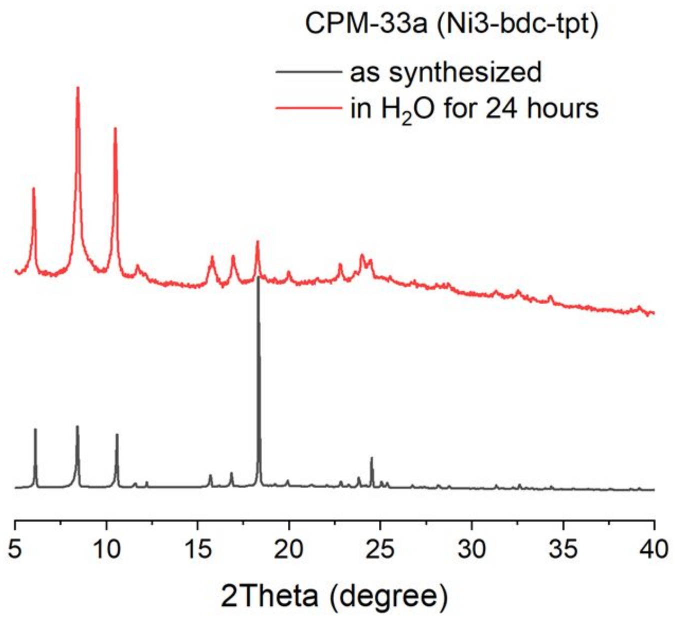

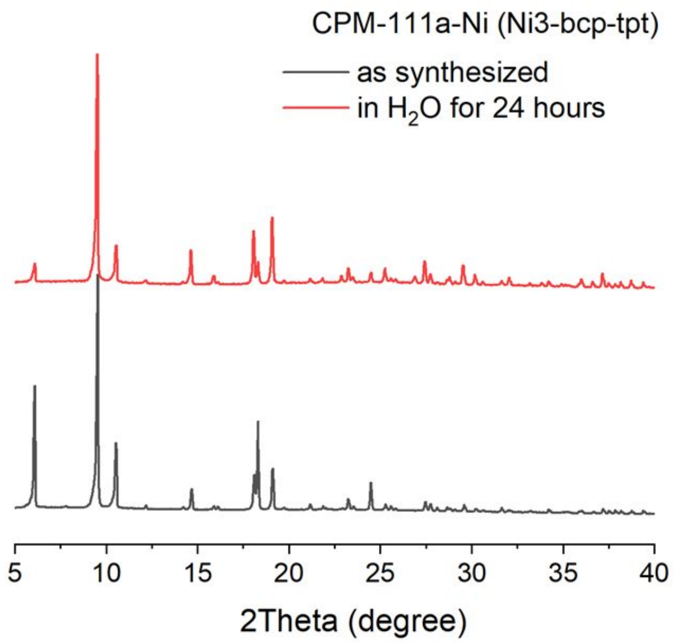

Figure S17. PXRD of Ni3-bdc-tpt (CPM-33a) and Ni3-bcp-tpt (CPM-111a-Ni) before and after in H_2O for 24 hours. The samples after gas adsorptions were used for water treatments so that their gas adsorption isotherms can be directly compared.

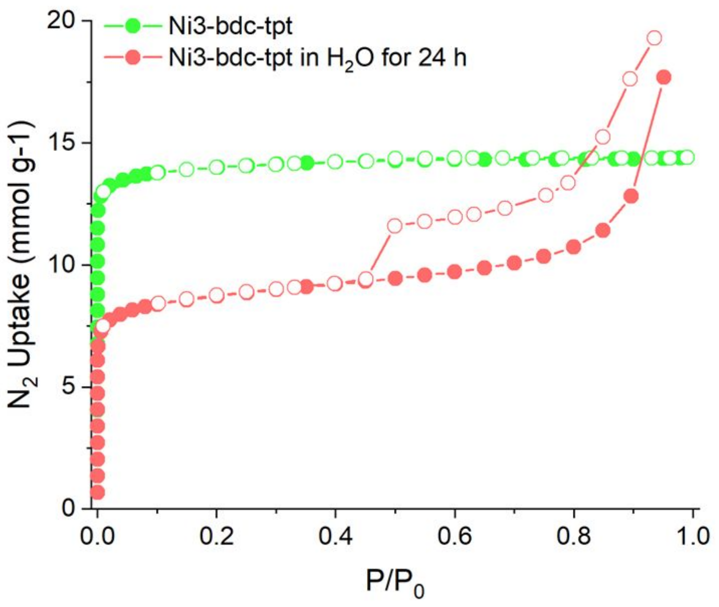

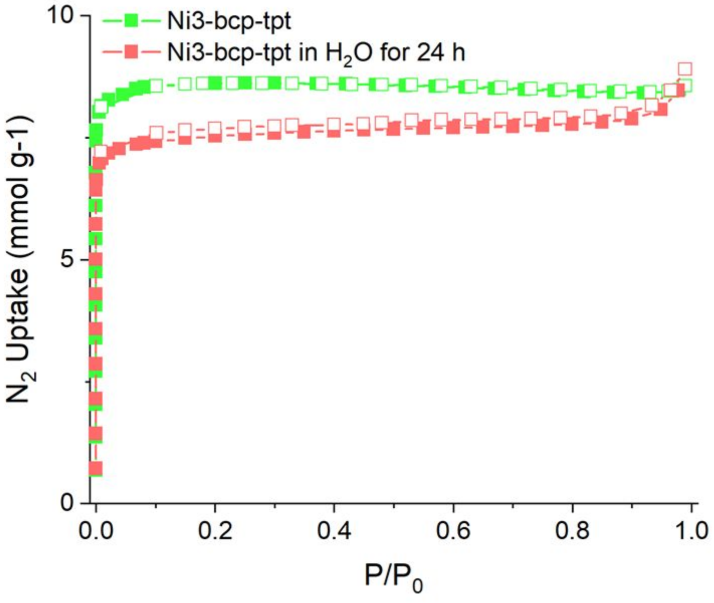

Figure S18. N_2 adsorption at 77 K of Ni3-bdc-tpt (CPM-33a) and Ni3-bcp-tpt (CPM-111a-Ni) before and after in H_2O for 24 hours.

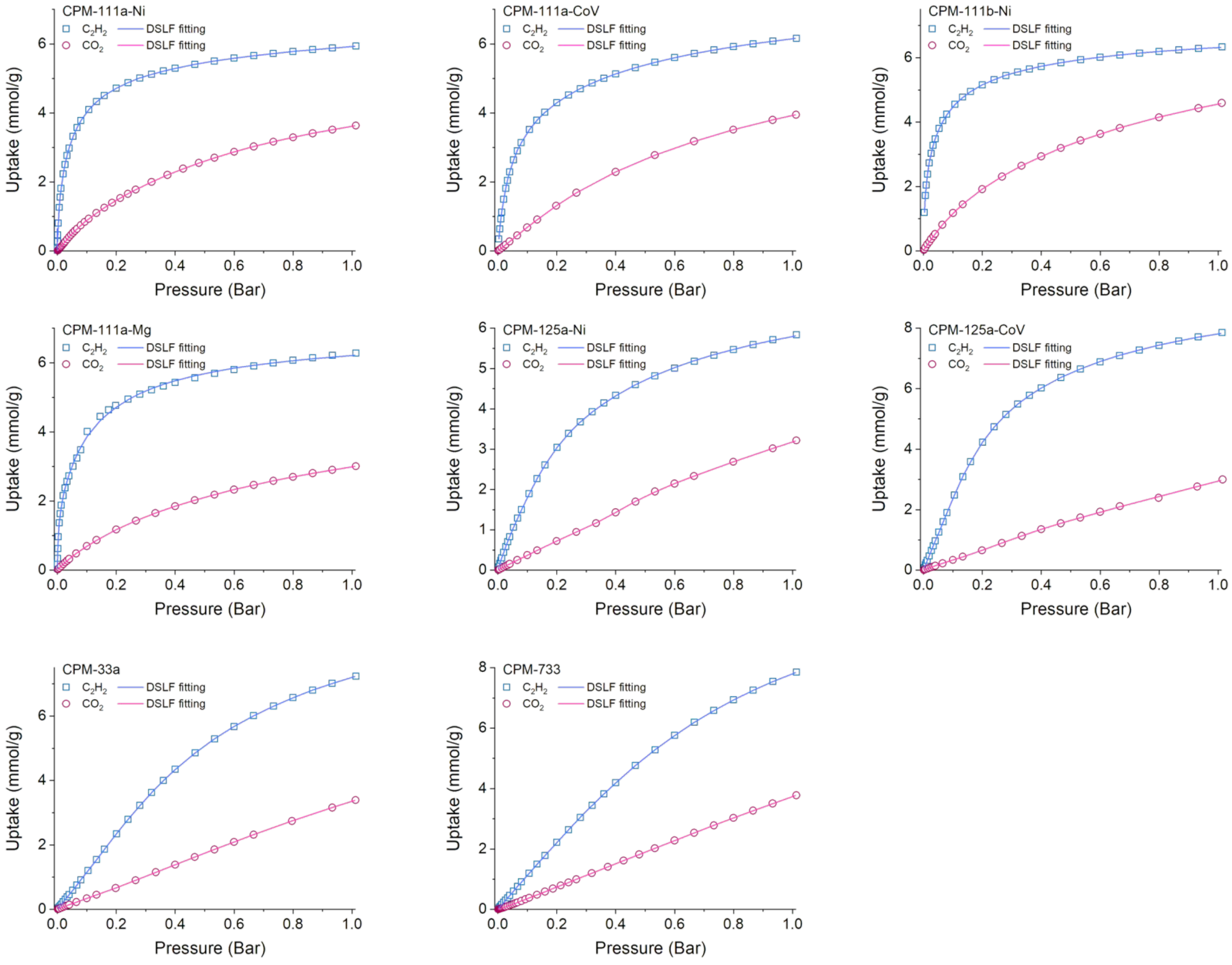

Figure S19. DSLF fitting of C_2H_2 and CO_2 adsorption isotherms of all MOFs reported in this work

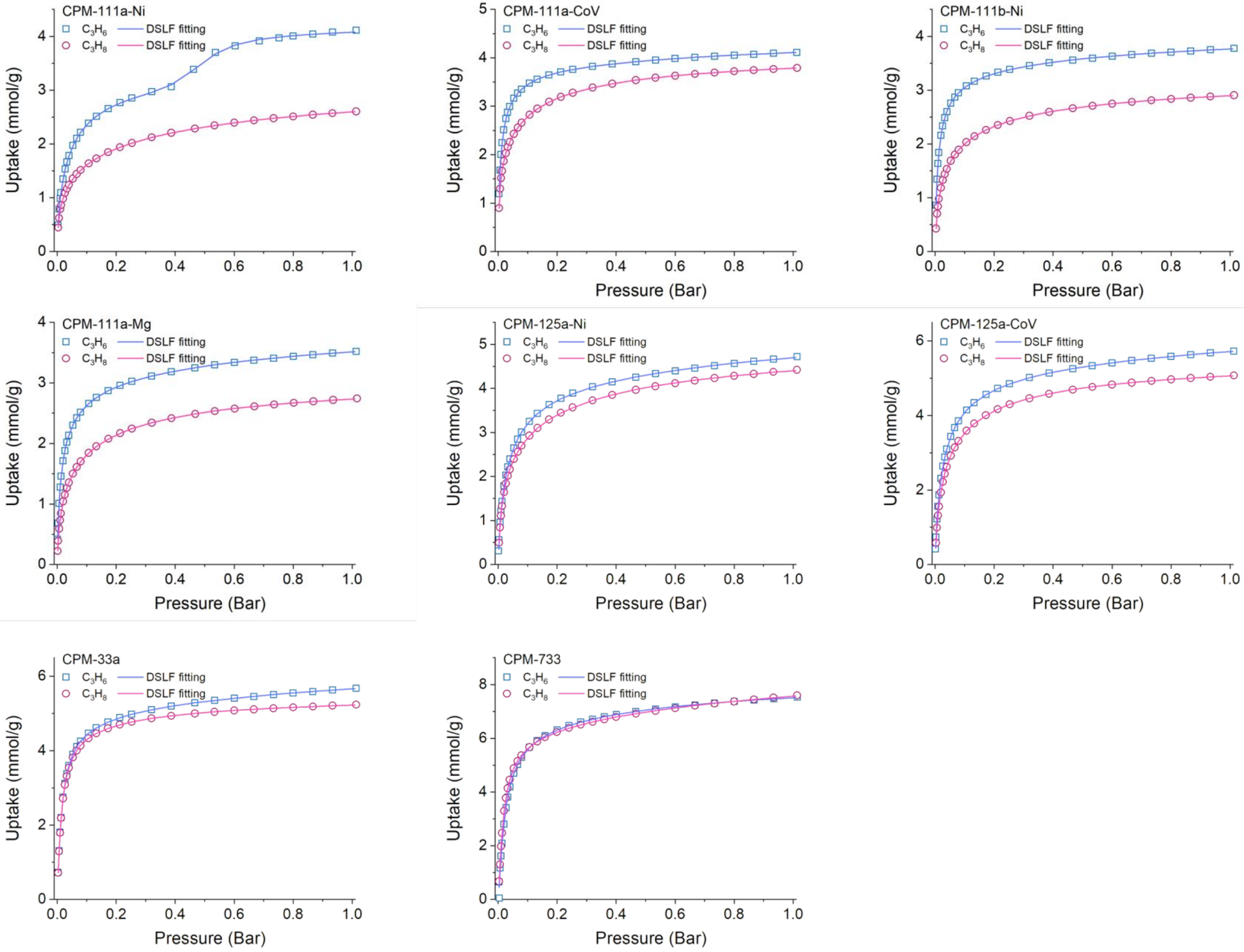

Figure S20. DSLF fitting of C_3H_6 and C_3H_8 adsorption isotherms of all MOFs reported in this work.

Table S1. The numbering scheme and names for the MOFs reported in this work

Numbering Code	Metal trimers	Dicarboxylic ligand	Partitioning Ligand	Composition code
CPM-111a-Ni	Ni3	bcp	tpt	Ni3-bcp-tpt
CPM-111a-CoV	Co_xV_{3-x}	bcp	tpt	CoV-bcp-tpt
CPM-111a-NiV	Ni_xV_{3-x}	bcp	tpt	NiV-bcp-tpt
CPM-111a-Mg	Mg3	bcp	tpt	Mg3-bcp-tpt
CPM-111a-Co	Co3	bcp	tpt	Co3-bcp-tpt
CPM-111b-Ni	Ni3	bcp	tppy	Ni3-bcp-tppy
CPM-125a-Ni	Ni3	cdc	tpt	Ni3-cdc-tpt
CPM-125a-CoV	Co_xV_{3-x}	cdc	tpt	CoV-cdc-tpt
CPM-125b-Ni	Ni3	Cdc	tppy	Ni3-cdc-tppy
CPM-33a[S1]	Ni3	bdc	tpt	Ni3-bdc-tpt
CPM-733[S4]	Co_2V	bdc	tpt	CoV-bdc-tpt

Table S2. Crystal data and structure refinement for pacs-Ni3-cdc-tpt

Identification code	Ni3-cdc-tpt	
Empirical formula	C48 H30 N6 Ni3 O13	
Formula weight	1074.91	
Temperature	296(2) K	
Wavelength	0.71073 Å	
Crystal system	Hexagonal	
Space group	$P6_3/mmc$	
Unit cell dimensions	a = 16.857(4) Å	α= 90°.
	b = 16.857(4) Å	β= 90°.
	c = 14.734(4) Å	γ = 120°.
Volume	3626(2) $Å^3$	
Z	2	
Density (calculated)	0.985 Mg/m^3	
Absorption coefficient	0.818 mm^{-1}	
F(000)	1096	
Theta range for data collection	1.964 to 27.495°.	
Index ranges	-21<=h<=21, -16<=k<=21, -18<=l<=18	
Reflections collected	25517	
Independent reflections	1585 [R(int) = 0.0716]	
Completeness to theta = 25.242°	99.9 %	
Refinement method	Full-matrix least-squares on F^2	
Data / restraints / parameters	1585 / 0 / 71	
Goodness-of-fit on F^2	1.272	
Final R indices [I>2sigma(I)]	R1 = 0.0399, wR2 = 0.1443	
R indices (all data)	R1 = 0.0547, wR2 = 0.1509	
Largest diff. peak and hole	0.470 and -0.688 e.$Å^{-3}$	

Table S3. Crystal data and structure refinement for pacs-Ni3-bcp-tpt

Identification code	Ni-bcp-tpt	
Empirical formula	C39 H30 N6 Ni3 O13	
Formula weight	322.27	
Temperature	296(2) K	
Wavelength	0.71073 Å	
Crystal system	Hexagonal	
Space group	$P6_3/mmc$	
Unit cell dimensions	a = 16.950(19) Å	α= 90°.
	b = 16.950(19) Å	β= 90°.
	c = 12.062(14) Å	γ = 120°.
Volume	3001(7) $Å^3$	
Z	2	
Density (calculated)	1.070 Mg/m^3	
Absorption coefficient	0.981 mm^{-1}	
F(000)	988	
Theta range for data collection	2.185 to 25.241°.	
Index ranges	-20<=h<=20, -20<=k<=19, -14<=l<=14	
Reflections collected	18346	
Independent reflections	1061 [R(int) = 0.0391]	
Completeness to theta = 25.241°	99.9 %	
Refinement method	Full-matrix least-squares on F^2	
Data / restraints / parameters	1061 / 77 / 83	
Goodness-of-fit on F^2	1.273	
Final R indices [I>2sigma(I)]	R1 = 0.0626, wR2 = 0.1522	
R indices (all data)	R1 = 0.0647, wR2 = 0.1532	
Largest diff. peak and hole	0.841 and -0.849 e.$Å^{-3}$	

Table S4. Crystal data and structure refinement for pacs-Mg3-bcp-tpt.

Identification code	Mg3-bcp-tpt	
Empirical formula	C39 H30 Mg3 N6 O13	
Formula weight	863.62	
Temperature	296(2) K	
Wavelength	0.71073 Å	
Crystal system	Hexagonal	
Space group	$P6_3/mmc$	
Unit cell dimensions	a = 16.910(8) Å	α= 90°.
	b = 16.910(8) Å	β= 90°.
	c = 12.375(6) Å	γ = 120°.
Volume	3065(3) $Å^3$	
Z	2	
Density (calculated)	0.936 Mg/m^3	
Absorption coefficient	0.098 mm^{-1}	
F(000)	892	
Theta range for data collection	1.390 to 25.409°.	
Index ranges	-19<=h<=16, -19<=k<=18, -14<=l<=14	
Reflections collected	9547	
Independent reflections	1098 [R(int) = 0.1081]	
Completeness to theta = 25.242°	100.0 %	
Refinement method	Full-matrix least-squares on F^2	
Data / restraints / parameters	1098 / 0 / 71	
Goodness-of-fit on F^2	0.984	
Final R indices [I>2sigma(I)]	R1 = 0.0538, wR2 = 0.1456	
R indices (all data)	R1 = 0.0873, wR2 = 0.1660	
Largest diff. peak and hole	0.574 and -0.272 e.$Å^{-3}$	

Table S5. Crystal data and structure refinement for pacs-Co3-bcp-tpt.

Identification code	Co3-bcp-tpt	
Empirical formula	C39 H30 Co3 N6 O13	
Formula weight	967.48	
Temperature	296(2) K	
Wavelength	0.71073 Å	
Crystal system	Hexagonal	
Space group	$P6_3/mmc$	
Unit cell dimensions	a = 16.866(5) Å	α= 90°.
	b = 16.866(5) Å	β= 90°.
	c = 12.601(4) Å	γ = 120°.
Volume	3104.1(19) $Å^3$	
Z	2	
Density (calculated)	1.035 Mg/m^3	
Absorption coefficient	0.840 mm^{-1}	
F(000)	982	
Theta range for data collection	2.135 to 25.424°.	
Index ranges	-15<=h<=15, -20<=k<=14, -15<=l<=15	
Reflections collected	9702	
Independent reflections	1118 [R(int) = 0.0942]	
Completeness to theta = 25.242°	99.9 %	
Refinement method	Full-matrix least-squares on F^2	
Data / restraints / parameters	1118 / 21 / 71	
Goodness-of-fit on F^2	1.039	
Final R indices [I>2sigma(I)]	R1 = 0.0410, wR2 = 0.1078	
R indices (all data)	R1 = 0.0603, wR2 = 0.1177	
Largest diff. peak and hole	0.419 and -0.420 e.$Å^{-3}$	

Table S6. Comparison of gas adsorption properties of MOFs reported in this work.

MOF compositions			Ni3-bcp-tpt	Co2V-bcp-tpt	Mg3-bcp-tpt	Ni3-bcp-tppy	Ni3-cdc-tpt	Co2V-cdc-tpt	Ni3-bdc-tpt	Co2V-bdc-tpt[S4,5]
BET/Langmuir surface area (m^2/g)			741.8.3/800.7	977.3/1074.8	295.8/308.5.	774.2/806.9	1221.6/1441.4	1404.6/1527.7	1268.7/1378.8	1328.5/1501.7
DFT pore size (Å)[b]			5.9	6.8	N.A.[d]	5.9	5.9	5.9	6.8	7.3
C_2H_2	Uptake, 298 K, 1 atm	Gravimetric (cm^3/g)	133.0	144.1	140.7	141.9	130.7	175.9	162.1	176
		Volumetric (cm^3/ cm^3)	142.3	N.A.	131.7	N.A.	128.7	N.A.	144.9	157
	273 K, 1atm (cm^3/g)		145.7	169.1	151.6	154.2	158.7	212.4	210.7	251
	Q^0_{st} (-kJ/mol)		37.5	42.8	46.4	45.4	33.5	29.8	30.7	22.9
CO_2	298 K, 1 atm (cm^3/g)		81.3	88.4	67.3	102.9	72.0	67.0	76.0	87
	273 K, 1atm (cm^3/g)		108.0	121.2	110.2	126.2	130.7	128.8	142.0	171
	Q^0_{st} (-kJ/mol)		37.4	29.3	35.8	33.9	27.3	19.1	22.4	23.8
C_2H_2/CO_2 Selectivity (50/50)[a]			20.1	8.6	29.0	14.7	4.6	6.6	3.4	2.9
Separation Potential (mmol/g)			5.0	4.4	5.3	5.2	3.4	5.2	3.2	3.1

C_3H_6 uptake at 298 K, 1 atm (cm^3/g)	92.0	92.1	78.8	84.6	105.7	128.3	127.1	168.7
C_3H_8 uptake at 298 K, 1atm	58.3	84.9	61.4	65.0	99.1	113.7	117.3	170.2
C_3H_6/C_3H_8 selectivity	3.2	2.6	4.2	5.8	1.3	1.7	1.2	0.84

a: The selectivity at 298 K and 1 bar is used; b: The pore size refers to the peak position from DFT pore size distribution. c. N.T.: not tested; d: the pore size of 111a-Mg is not available due to the small number of adsorption points at low pressure.

Table S7. Summary for benchmark MOFs for highly selective C_2H_2/CO_2 separation with selectivity higher than 12. The MOFs are arranged in descending sequence of gravimetric C_2H_2 uptake.

MOFs	C_2H_2 Uptake (STP) at 0.1 bar	C_2H_2 Uptake (STP) at 1 atm		Q^0_{st} (-kJ/mol)	Selectivity (STP, 50/50)	Separation Potential (mmol/g)	Density (g/cm^3)[b]	Reference
		Gravimetric	Volumetric					
**CPM-111b-Ni**	**4.47**	**141.9**	**N.A.**	**45.4**	**14.7**	**5.2**	**N.A.**	**This work**
**CPM-111a-Mg**	**4.01**	**140.7**	**131.7**	**46.4**	**29.0**	**5.3**	**0.936**	**This work**
**CPM-111a-Ni**	**4.02**	**133.0**	**142.3**	**37.5**	**20.1**	**5.0**	**1.070**	**This work**
ATC-Cu	3.83	112.2	158.1	79.1	53.6	N.A.	1.409	[S6]
FeNi-M'MOF	3.37	96.1	133	27	24	3.76	1.375	[S7]
ZJU-74a	3.39	85.7	116.0	45	36.5	3.06	1.353	[S8]
BSF-3	2.22	81.8	77.7	42.7	16.3	2.78	0.95	[S9]
JCM-1	1.83	75	99.8	36.9	13.7	2.43	1.331	[S10]
UTSA-300	1.34	68.9	107.6	57.6	743	N.A.	1.562	[S11]
MOF-OH	1.38	68.2	107.7	17.5	25	N.A.	1.579	[S12]
$Mg(HCOO)_2$	1.98	66	91	>45	12.7[c]	N.A.	1.379	[S13,14]
NKMOF-1-Ni	2.12	61.0	109.8	60.3	20.5	N.A.	1.800	[S15]

$Zn_2(bpy)(btec)$[d]	1.16	51.5	93.5	28.7	33.3	N.A.	1.692	S16
Cu^I@UiO-66-$(COOH)_2$	1.34	51.7	N.A.	74.5	185	2.89	N.A.	S17
DICRO-4-Ni-i	1.07	43	52.2	37.7	13.9	1.43	1.215	S18

a: The uptake at 0.1 bar is either calculated based on the fitting equations or by digitalizing the adsorption isotherm.

b: The density is calculated based on the cif file with any solvent or gas being removed. The removal of solvent or gas is based on the fact that the activated materials for gas adsorption have no solvents or gas inside.

c: The selectivity value of $Mg(HCOO)_2$ is derived from the Henry constant calculation for this temperature.

d: The discrepancy was found in the density calculated from the cif file and from ratio of volumetric uptake to gravimetric uptake reported in the reference.

Table S8. Summary for benchmark MOFs for C_2H_2/CO_2 separation with uptakes comparable or higher than CPM-111a-Ni. The MOFs are arranged in descending sequence of selectivity.

MOFs	C_2H_2 Uptake (STP) at	Selectivity (STP, 50/50)	C_2H_2 Uptake (STP)		Q^0_{st} (-kJ/mol)	Separation Potential (mmol/g)	Density (g/cm^3)[a]	Reference
			Gravimetric (cm^3/g)	Volumetric (cm^3/cm^3)				
**CPM-111a-Mg**	**4.01**	**29.0**	**140.7**	**131.7**	**46.4**	**5.3**	**0.936**	**This work**
**CPM-111a-Ni**	**4.02**	**20.1**	**133.0**	**142.3**	**37.5**	**5.0**	**1.070**	**This work**
**CPM-111b-Ni**	**4.47**	**14.7**	**141.9**	N.A.	**45.4**	**5.2**	**N.A.**	**This work**
MIL-160	3.74	10	191	213	31.8	6.0	1.1	S19
UTSA-74	2.54	9	108.2[b]	145	31	3.41	1.34	S20
FJI-H8-Me	3.13	5.7	229	200.3	33.7	5.5[a]	0.875	S21
NBU-8	1.21	5.4	182.9	170.6	34.6	N.A.	0.933	S22
Cu-TPA	5.06	5.3	185	184.1	39.1	N.A.	0.995	S23
SNNU-45	2.23	4.5	134	113.1	40	N.A.	0.844	S24
FJU-90	2.68	4.3	180	146.9	25.1	4.5[a]	0.816	S25
CAU-10-H	1.11	4.0	89.8	100.6	~27	1.56	1.12	S26
SNNU-27-Fe	0.96	2.0	182.4	N.A.	24.1	<1.0	N.A.	S27

a: The value is calculated by digitalizing the graph. B. The value is calculated through volumetric uptake and the density of UTSA-74a

Table S9. Summary of the refined parameters with Dual-Site Langmuir-Freundlich fitting for gas adsorption isotherms

**MOFs**	Gases	A_1 (mmol g^{-1})	B_1 (bar$^{-1/n1}$)	$1/n_1$	A_2 (mmol g^{-1})	B_2 (bar$^{-1/n2}$)	$1/n_2$	R^2
**CPM-111a-Ni Ni3-bcp-tpt**	C_2H_2	6.48703	8.23521	0.70416	0.38471	0.58055	2027086	0.99998
	CO_2	4.69523	1.52083	1.20731	0.81427	23.56205	1.21380	>0.99999[a]
	C_3H_6	3.65444	9.16574	0.70487	0.78052	606.332	8.92402	0.99981
	C_3H_8	2.64084	2.03008	0.63560	0.83953	72.3910	0.85304	>0.99999
**CPM-111b-Ni Ni3-bcp-tppy**	C_2H_2	7.18245	6.26768	0.60956	0.11897	1325060	2.61527	0.99998
	CO_2	8.18733	1.14733	0.89246	0.18659	330.646	2.29232	>0.99999
	C_3H_6	1.58349	1.27151	0.54025	2.91352	78.1474	0.93495	0.99998
	C_3H_8	2.50606	4.19124	0.65806	0.88426	88.5875	0.98228	>0.99999
**CPM-111a-CoV CoV-bcp-tpt**	C_2H_2	4.52276	19.15969	0.95105	3.05733	1.53178	1.07827	>0.99999
	CO_2	0.10225	197.113	3.21438	7.00423	1.20148	1.05369	>0.99999
	C_3H_6	0.51346	0.79011	1.24908	3.98675	36.8406	0.77717	>0.99999
	C_3H_8	2.99789	6.06708	0.72845	1.21314	1100.58	1.19812	>0.99999
**CPM-111a-Mg Mg3-bcp-tpt**	C_2H_2	5.83495	6.107	0.86049	1.19338	1643.48	1.22293	0.99932
	CO_2	4.45086	0.85145	1.01679	1.19217	3.77602	0.94925	>0.99999
	C_3H_6	2.93285	28.9764	0.80731	1.75559	0.63106	0.62839	0.99999
	C_3H_8	0.89991	46.6557	0.87963	2.34593	3.76923	0.67797	0.99999
**CPM-125a-Ni Ni3-cdc-tpt**	C_2H_2	7.29054	2.77010	0.95882	0.43883	589.259	3.31963	0.99998
	CO_2	14.39640	0.26076	0.99482	0.20306	3950.52	9.96117	0.99998
	C_3H_6	2.66862	0.48115	0.67782	4.03401	18.7534	0.83549	0.99993
	C_3H_8	4.06018	3.35596	0.67387	1.27946	188.063	1.16825	0.99997
**CPM-125a-CoV CoV-cdc-tpt**	C_2H_2	1.73783	103.952	2.44120	8.17535	2.92099	0.98736	0.99999
	CO_2	22217.5	0.000122	0.91260	0.22117	195.734	4.43803	0.99986
	C_3H_6	2.80297	0.44348	0.68644	5.06504	23.1262	0.86414	0.99999
	C_3H_8	3.98783	5.82682	0.74895	1.67568	100.403	1.08117	0.99999

**CPM-33a Ni3-bdc-tpt**	C_2H_2	3.14764	6.58061	1.96083	8.43478	1.12847	0.95606	>0.99999
	CO_2	0.17050	33.93321	1.14966	9.71562	0.48892	1.32942	>0.99999
	C_3H_6	4.03783	146.484	1.19717	4.90891	0.50701	0.42833	0.99999
	C_3H_8	2.22224	3.01011	0.56811	3.57372	218.566	1.26371	0.99999
**CPM-733 Co2V-bdc-tpt**	C_2H_2	5.28437	2.11031	0.99831	6.39745	1.93489	1.77337	>0.99999
	CO_2	1.53444	2.49538	1.08306	9.65928	0.37583	1.59810	>0.99999
	C_3H_6	5.73030	148.178	1.31020	2.64614	2.17397	0.92405	0.99834
	C_3H_8	4.71097	325.213	1.42007	7.03738	0.68984	0.50399	0.99999

a: >0.99999 indicates the R^2 is at least 0.999995.

Reference

S1. Zhao, X.; Bu, X.; Zhai, Q.-G.; Tran, H.; Feng, P. Pore Space Partition by Symmetry-Matching Regulated Ligand Insertion and Dramatic Tuning on Carbon Dioxide Uptake. *Journal of the American Chemical Society* **2015**, *137*, 1396.

S2. Myers, A. L.; Prausnitz, J. M. Thermodynamics of mixed-gas adsorption. *AlChE J.* **1965**, *11*, 121.

S3. Krishna, R. Screening metal–organic frameworks for mixture separations in fixed-bed adsorbers using a combined selectivity/capacity metric. *RSC Advances* **2017**, *7*, 35724.

S4. Yang, H.; Wang, Y.; Krishna, R.; Jia, X.; Wang, Y.; Hong, A. N.; Dang, C.; Castillo, H. E.; Bu, X.; Feng, P. Pore-Space-Partition-Enabled Exceptional Ethane Uptake and Ethane-Selective Ethane–Ethylene Separation. *Journal of the American Chemical Society* **2020**, *142*, 2222.

S5. Wang, Y.; Jia, X.; Yang, H.; Wang, Y.; Chen, X.; Hong, A. N.; Li, J.; Bu, X.; Feng, P. A Strategy for Constructing Pore-Space-Partitioned MOFs with High Uptake Capacity for C2 Hydrocarbons and CO2. *Angewandte Chemie International Edition* **2020**, *59*, 19027.

S6. Niu, Z.; Cui, X.; Pham, T.; Verma, G.; Lan, P. C.; Shan, C.; Xing, H.; Forrest, K. A.; Suepaul, S.; Space, B.; Nafady, A.; Al-Enizi, A. M.; Ma, S. A MOF-based Ultra-Strong Acetylene Nano-trap for Highly Efficient C2H2/CO2 Separation. *Angewandte Chemie International Edition* **2021**, *60*, 5283.

S7. Gao, J.; Qian, X.; Lin, R.-B.; Krishna, R.; Wu, H.; Zhou, W.; Chen, B. Mixed Metal–Organic Framework with Multiple Binding Sites for Efficient C2H2/CO2 Separation. *Angewandte Chemie International Edition* **2020**, *59*, 4396.

S8. Pei, J.; Shao, K.; Wang, J.-X.; Wen, H.-M.; Yang, Y.; Cui, Y.; Krishna, R.; Li, B.; Qian, G. A Chemically Stable Hofmann-Type Metal−Organic Framework with Sandwich-Like Binding Sites for Benchmark Acetylene Capture. *Advanced Materials* **2020**, *32*, 1908275.

S9. Zhang, Y.; Hu, J.; Krishna, R.; Wang, L.; Yang, L.; Cui, X.; Duttwyler, S.; Xing, H. Rational Design of Microporous MOFs with Anionic Boron Cluster Functionality and Cooperative Dihydrogen Binding Sites for Highly Selective Capture of Acetylene. *Angewandte Chemie International Edition* **2020**, *59*, 17664.

S10. Lee, J.; Chuah, C. Y.; Kim, J.; Kim, Y.; Ko, N.; Seo, Y.; Kim, K.; Bae, T. H.; Lee, E. Separation of Acetylene from Carbon Dioxide and Ethylene by a Water-Stable Microporous Metal–Organic Framework with Aligned Imidazolium Groups inside the Channels. *Angewandte Chemie International Edition* **2018**, *57*, 7869.

S11. Lin, R.-B.; Li, L.; Wu, H.; Arman, H.; Li, B.; Lin, R.-G.; Zhou, W.; Chen, B. Optimized Separation of Acetylene from Carbon Dioxide and Ethylene in a Microporous Material. *Journal of the American Chemical Society* **2017**, *139*, 8022.

S12. Gong, W.; Cui, H.; Xie, Y.; Li, Y.; Tang, X.; Liu, Y.; Cui, Y.; Chen, B. Efficient C2H2/CO2 Separation in Ultramicroporous Metal–Organic Frameworks with Record C2H2 Storage Density. *Journal of the American Chemical Society* **2021**, *143*, 14869.

S13. Fischer, M.; Hoffmann, F.; Fröba, M. New Microporous Materials for Acetylene Storage and C2H2/CO2 Separation: Insights from Molecular Simulations. *ChemPhysChem* **2010**, *11*, 2220.

S14. Samsonenko , D. G.; Kim, H.; Sun, Y.; Kim, G.-H.; Lee, H.-S.; Kim, K. Microporous Magnesium and Manganese Formates for Acetylene Storage and Separation. *Chemistry – An Asian Journal* **2007**, *2*, 484.

S15. Peng, Y.-L.; Pham, T.; Li, P.; Wang, T.; Chen, Y.; Chen, K.-J.; Forrest, K. A.; Space, B.; Cheng, P.; Zaworotko, M. J.; Zhang, Z. Robust Ultramicroporous Metal–Organic Frameworks with Benchmark Affinity for Acetylene. *Angewandte Chemie International Edition* **2018**, *57*, 10971.

S16. Chen, Y.; Du, Y.; Wang, Y.; Krishna, R.; Li, L.; Yang, J.; Li, J.; Mu, B. A stable metal–organic framework with well-matched pore cavity for efficient acetylene separation. *AIChE Journal* **2021**, *67*, e17152.

S17. Zhang, L.; Jiang, K.; Yang, L.; Li, L.; Hu, E.; Yang, L.; Shao, K.; Xing, H.; Cui, Y.; Yang, Y.; Li, B.; Chen, B.; Qian, G. Benchmark C2H2/CO2 Separation in an Ultra-Microporous Metal–Organic Framework via Copper(I)-Alkynyl Chemistry. *Angewandte Chemie International Edition* **2021**, *60*, 15995.

S18. Scott, H. S.; Shivanna, M.; Bajpai, A.; Madden, D. G.; Chen, K.-J.; Pham, T.; Forrest, K. A.; Hogan, A.; Space, B.; Perry Iv,

J. J.; Zaworotko, M. J. Highly Selective Separation of C2H2 from CO2 by a New Dichromate-Based Hybrid Ultramicroporous Material. *ACS Applied Materials & Interfaces* **2017**, *9*, 33395.
S19. Ye, Y.; Xian, S.; Cui, H.; Tan, K.; Gong, L.; Liang, B.; Pham, T.; Pandey, H.; Krishna, R.; Lan, P. C.; Forrest, K. A.; Space, B.; Thonhauser, T.; Li, J.; Ma, S. Metal–Organic Framework Based Hydrogen-Bonding Nanotrap for Efficient Acetylene Storage and Separation. *Journal of the American Chemical Society* **2021**.
S20. Luo, F.; Yan, C.; Dang, L.; Krishna, R.; Zhou, W.; Wu, H.; Dong, X.; Han, Y.; Hu, T.-L.; O'Keeffe, M.; Wang, L.; Luo, M.; Lin, R.-B.; Chen, B. UTSA-74: A MOF-74 Isomer with Two Accessible Binding Sites per Metal Center for Highly Selective Gas Separation. *Journal of the American Chemical Society* **2016**, *138*, 5678.
S21. Di, Z.; Liu, C.; Pang, J.; Chen, C.; Hu, F.; Yuan, D.; Wu, M.; Hong, M. Cage-Like Porous Materials with Simultaneous High C2H2 Storage and Excellent C2H2/CO2 Separation Performance. *Angewandte Chemie International Edition* **2021**, *60*, 10828.
S22. Li, Q.; Wu, N.; Li, J.; Wu, D. A Highly Connected Trinuclear Cluster Based Metal ‒ Organic Framework for Efficient Separation of C2H2/C2H4 and C2H2/CO2. *Inorganic Chemistry* **2020**, *59*, 13005.
S23. Li, H.; Liu, C.; Chen, C.; Di, Z.; Yuan, D.; Pang, J.; Wei, W.; Wu, M.; Hong, M. An Unprecedented Pillar-Cage Fluorinated Hybrid Porous Framework with Highly Efficient Acetylene Storage and Separation. *Angewandte Chemie International Edition* **2021**, *60*, 7547.
S24. Li, Y.-P.; Wang, Y.; Xue, Y.-Y.; Li, H.-P.; Zhai, Q.-G.; Li, S.-N.; Jiang, Y.-C.; Hu, M.-C.; Bu, X. Ultramicroporous Building Units as a Path to Bi-microporous Metal–Organic Frameworks with High Acetylene Storage and Separation Performance. *Angewandte Chemie International Edition* **2019**, *58*, 13590.
S25. Ye, Y.; Ma, Z.; Lin, R.-B.; Krishna, R.; Zhou, W.; Lin, Q.; Zhang, Z.; Xiang, S.; Chen, B. Pore Space Partition within a Metal–Organic Framework for Highly Efficient C2H2/CO2 Separation. *Journal of the American Chemical Society* **2019**, *141*, 4130.
S26. Pei, J.; Wen, H.-M.; Gu, X.-W.; Qian, Q.-L.; Yang, Y.; Cui, Y.; Li, B.; Chen, B.; Qian, G. Dense Packing of Acetylene in a Stable and Low-Cost Metal–Organic Framework for Efficient C2H2/CO2 Separation. *Angewandte Chemie International Edition* **2021**, *60*, 25068.
S27. Xue, Y.-Y.; Bai, X.-Y.; Zhang, J.; Wang, Y.; Li, S.-N.; Jiang, Y.-C.; Hu, M.-C.; Zhai, Q.-G. Precise Pore Space Partitions Combined with High-Density Hydrogen-Bonding Acceptors within Metal–Organic Frameworks for Highly Efficient Acetylene Storage and Separation. *Angewandte Chemie International Edition* **2021**, *60*, 10122.
